# The Road Less Traveled: Exploring the Genomic Characteristics and Antimicrobial Resistance Potential of *Acinetobacter baumannii* From the Indigenous Orang Asli Community in Peninsular Malaysia

**DOI:** 10.1002/mbo3.70073

**Published:** 2025-10-17

**Authors:** Soo‐Sum Lean, Denise E. Morris, Rebecca Anderson, Ahmed Ghazi Alattraqchi, David W. Cleary, Stuart C. Clarke, Chew Chieng Yeo

**Affiliations:** ^1^ Faculty of Medicine and Institute for Life Sciences University of Southampton Southampton UK; ^2^ Centre for Research in Infectious Diseases and Biotechnology, Faculty of Medicine Universiti Sultan Zainal Abidin Kuala Terengganu Malaysia; ^3^ Department of Microbes, Infections and Microbiomes, School of Infection, Inflammation and Immunology, College of Medicine and Health University of Birmingham Birmingham UK; ^4^ Institute of Microbiology and Infection University of Birmingham Birmingham UK; ^5^ NIHR Southampton Biomedical Research Centre University Hospital Southampton Foundation NHS Trust Southampton UK; ^6^ Global Health Research Institute University of Southampton Southampton UK; ^7^ Institute for Research, Development and Innovation International Medical University Kuala Lumpur Malaysia; ^8^ Department of Biological Sciences, Faculty of Science Universiti Tunku Abdul Rahman Kampar Malaysia

**Keywords:** antibiotic resistance genes, indigenous community *Acinetobacter baumannii*, Orang Asli, p*dif* modules, plasmids, virulence factors

## Abstract

*Acinetobacter baumannii* is widely recognized as a multidrug‐resistant pathogen, although most public genome datasets are biased toward hospital‐derived isolates. Little is known about *A. baumannii* isolates from healthy individuals in the community. This study analyzed genome sequences from nine *A. baumannii* isolates obtained from the upper respiratory tract of the indigenous Orang Asli in their rural community in Peninsular Malaysia. Genomic analysis revealed genetic diversity, including three new Pasteur sequence types (STs) and six novel Oxford STs. One isolate, *A. baumannii* 19064, belonged to Global Clone 8 (GC8), a lineage linked to clinical infections. Core genome phylogeny showed these community isolates interspersed with clinical isolates from a nearby hospital, indicating potential pathogenicity under suitable conditions. All isolates carried intrinsic *bla*_OXA‐51‐like_ carbapenemase and *bla*_ADC_ cephalosporinase genes but remained susceptible to meropenem. Two isolates, *A. baumannii* 19053 and 19062, were tetracycline resistant but minocycline susceptible, and harbored the *tet(39)–tetR* gene pair within a mobile p*dif* module on distinct Rep_3‐type plasmids. Only one isolate, *A. baumannii* 19055, is plasmid‐free; the rest mainly harbored cryptic plasmids, often containing identifiable p*dif* modules. These findings highlight the clinical relevance of *A. baumannii* strains residing in healthy individuals, particularly in isolated communities that are seldom accessible to public health. Despite their remote origins, these isolates possess virulence factors and resistance genes similar to those in hospital settings. This underscores the importance of genomic surveillance of commensal pathogens, as exploring these less‐studied isolates can yield insights into broader epidemiological trends and guide future public health strategies.

## Graphical abstract

Analysis of *Acinetobacter baumannii* from healthy individuals in a rural Malaysian community revealed significant genetic diversity, including novel sequence types. These isolates carry resistance genes and resemble clinical strains, highlighting their potential to cause disease despite their commensal origin. This underscores the need for genomic surveillance of community pathogens.

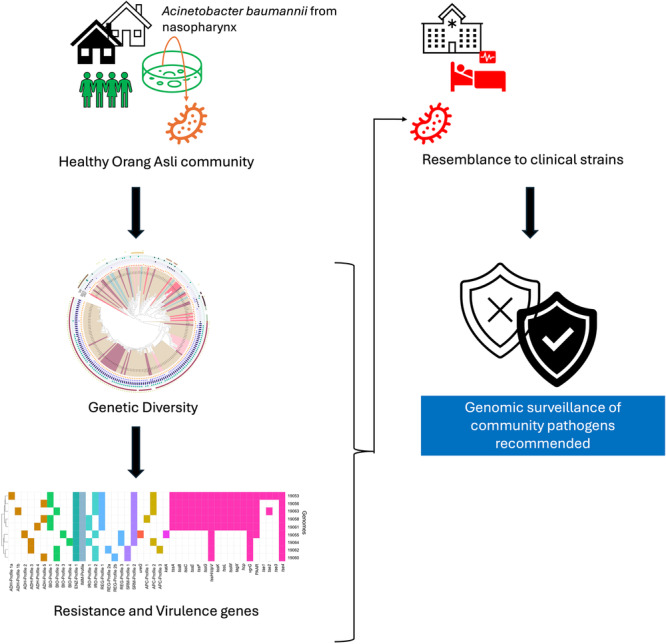

## 1 Introduction

Carbapenem‐resistant *Acinetobacter baumannii* is recognized by the World Health Organization (WHO) as the top critical priority pathogen, posing the highest threat to public health due to limited treatment options (World Health Organization 2024). Unsurprisingly, the majority of reported *A. baumannii* genomes and those deposited in public databases, such as NCBI Genomes and the PubMLST genome collection, are mainly of hospital origin. These isolates are obtained from patients with hospital‐acquired infections, such as ventilator‐associated pneumonia, meningitis, blood stream infections, and urinary tract infections (Bian et al. 2021; Cui et al. 2023; Shelenkov et al. 2024). As a result, the public *A. baumannii* genome datasets are heavily skewed toward dominant hospital‐associated clones, notably members of the notorious Global Clone 2 (GC2) lineage (Shelenkov et al. 2023). While keeping track of hospital‐related *A. baumannii* is essential due to their formidable antimicrobial resistance (AMR) and clinical relevance, they only represent a subset of the species. These strains often differ significantly from those found in the normal flora of healthy populations (Muzahid et al. 2023). *A. baumannii* from healthy communities remains largely understudied, more so for indigenous communities that are isolated from urban populations.

The Orang Asli are indigenous people in Peninsular Malaysia comprising several ethnic subgroups who retained their aboriginal language, customs and lifestyle (Mahmud et al. 2022). They are only a minor population in Malaysia (0.8% of the population in Peninsular Malaysia based on the year 2020 census) and often fall behind national socioeconomic, education and healthcare improvement plans. Despite government resettlement programs, many Orang Asli communities remain in rural areas due to their lifestyle preferences (Pah Rokiah Syed Hussain et al. 2017; Mahmud et al. 2022). Some of these isolated communities are difficult to reach, limiting their access to modern medicines, such as antibiotics and vaccines (Mohd Rosman et al. 2020; Chew et al. 2022). This restricted interaction with urban communities and healthcare has led to the formation of what we term here as “genomic capsules”—distinct microflora genomes unique to the Orang Asli community and their respective tribes. As a result, opportunistic pathogens such as *A. baumannii* harbored by the Orang Asli may differ from strains commonly present in urban hospitals.

*A. baumannii* from hospitals have been well‐studied over the past two decades, gaining their notoriety due to their multidrug resistance (MDR), extensive drug resistance, and pan‐drug resistance (PDR) characteristics (Shi et al. 2024). The Malaysian Ministry of Health has published annual National Surveillance of Antibiotic Resistance (NSAR) Reports since 2003. Beginning in the 2010s, more than 50% of *A. baumannii* isolates have been reported to be resistant to carbapenems (i.e., imipenem and meropenem), which are the drugs of choice for treatment. However, the NSAR data set is limited to participating hospitals, which are mainly in the urban and suburban areas of Malaysia (Ministry of Health 2023). While the WHO's Tracking AMR Country Self‐Assessment Survey (TrACSS) Country Report emphasizes the importance of addressing AMR at the community level to enhance infection prevention and control efforts (WHO 2022), there remains a significant knowledge gap regarding AMR profiles and genomic characteristics of bacterial pathogens within the indigenous communities in Malaysia. Notably, a recent study investigating *A. baumannii* isolates from human fecal samples in a community in Segamat, Malaysia, revealed phylogenomic clustering of four community‐derived strains with two isolates from the town's main tertiary hospital. This finding suggests the potential persistence and circulation of certain *A. baumannii* strains across both community and healthcare settings (Muzahid et al. 2023). In this study, we aim to provide a genomic snapshot of *A. baumannii* that were isolated during an all‐age, upper respiratory tract microbial carriage study undertaken among two rural Orang Asli communities in the state of Terengganu, located on the eastern coast of Peninsular Malaysia in 2017. A previous investigation of *Klebsiella pneumoniae* isolates from a broader indigenous cohort revealed the predominance of ST23, which is commonly associated with clinical *K. pneumoniae* infections, and of concern, a proportion of these isolates harbored genes that categorized them as hypervirulent (Das et al. 2024). Here, we present the genomic analysis of *A. baumannii* isolates recovered from the upper respiratory tract of the Orang Asli and show the genetic diversity of this hitherto unexplored *A. baumannii* “genomic capsule.” Our findings offer insights into strains of novel sequence types (STs), their patchwork of unique and shared mobile genetic elements, AMR, and virulence genes.

## 2 Materials and Methods

### 2.1 Sampling and Isolation of *A. baumannii* From Orang Asli Communities

Swabs were taken from two Orang Asli villages, namely, Kampung Sungai Pergam and Kampung Berua, in the state of Terengganu on the east coast of Peninsular Malaysia. Nasal swabs and nasopharyngeal swabs were taken from each participant, as described (Cleary et al. 2021). Conventional bacteriology of the samples were carried out using Columbia Blood Agar (CBA), CBA with chocolated horse blood agar, CBA with colistin and naladixic acid agar, CBA with chocolated horse blood and Bacitracin, and Lysed Gonococcal (GC) selective agar (all culture media from Oxoid, UK) (Cleary et al. 2021). Preliminary identification of presumptive *Acinetobacter* spp. isolates was done using the MALDI Biotyper (Bruker, UK) at the Portsmouth Microbiology Laboratories, UK.

### 2.2 Antibiotic Susceptibility Tests

*A. baumannii* was spread over Mueller‐Hinton agar plates (MH, Oxoid, UK). Susceptibilities to the antibiotics meropenem and ciprofloxacin were determined by disk diffusion using the appropriate antibiotic disk (meropenem, 10 µg; ciprofloxacin, 5 µg) (Oxoid, UK), whereas tetracycline and doxycycline susceptibilities were determined by placing minimum inhibitory concentration (MIC) E test strips (bioMérieux, France) onto the surface of the agar. All agar plates were incubated at 35°C ± 1°C for 18 ± 2 h. Susceptibility was determined against the EUCAST Clinical Breakpoint guidelines (2024).

### 2.3 Genome Sequencing and Assemblies

Genomic DNA of the nine *A. baumannii* isolates was extracted using the QIAmp DNA Mini extraction kit (Qiagen, UK) per the manufacturer's instructions. Concentration of genomic DNA was determined using a Qubit 2.0 fluorometer (Thermo‐Fisher, UK). Whole genome sequencing was performed on a MiSeq (Illumina, UK) short‐read platform at a commercial sequencing provider (MicrobesNG, UK) using the 500‐cycle v2 reagent kit to generate 2 × 150 bp paired‐end reads. Raw reads obtained were then quality assessed and trimmed using fastp (available from https://github.com/OpenGene/fastp; Chen 2023). Genome assemblies were carried out using Unicycler (available from https://github.com/rrwick/Unicycler; Wick et al. 2017), followed by evaluation using Quast (available from https://github.com/ablab/quast).

### 2.4 Bioinformatics Analyses

Average nucleotide identity (ANI) of the assembled genomes to reference genomes was determined using fastANI (available from https://github.com/ParBLiSS/FastANI; Jain et al. 2018). Annotation of the genomes was performed using Prokka (available from https://github.com/tseemann/prokka; Seemann 2014). Conventional multilocus sequence typing (MLST) profiles of the assembled genomes were determined using mlst (available from https://github.com/tseemann/mlst) and matched to the PubMLST database (https://pubmlst.org/organisms/acinetobacter-baumannii). MLST profiles determined from the two available schemes, namely, the Oxford and Pasteur schemes, were used to identify the corresponding Global Clones (GCs). Serotyping based on *A. baumannii* surface polysaccharide loci, namely, capsule K loci (KL) and lipo‐oligosaccharide OC loci (OCL), was carried out using Kaptive v3.0.0b6 (available from https://github.com/klebgenomics/Kaptive; Wyres et al. 2020; Cahill et al. 2022).

Genotypic resistance profiles of the genomes were determined using AMRFinderPlus (available from https://github.com/ncbi/amr; Feldgarden et al. 2021) and ABRicate (available from https://github.com/tseemann/abricate), whereby databases from CARD (Alcock et al. 2023) and ResFinder (Zankari et al. 2012) were utilized for the latter approach. Virulome of the assembled genomes was determined using ABRicate, utilizing database from Virulence Factor DataBase (Liu et al. 2022). Findings were then compared with the results obtained from VFAnalyzer (available from https://www.mgc.ac.cn/cgi-bin/VFs/v5/main.cgi). Mobile genetic elements such as plasmids, insertion sequence (IS) elements, and resistance island (RI) hotspots were also determined from the genomes. Plasmids were identified using PlasmidFinder (available from https://github.com/genomicepidemiology/plasmidfinder; Carattoli et al. 2014), whereas classification of the plasmid replication protein (Rep) was performed using an in‐house built script, pREPonly (https://github.com/lean-SS/pREP-only), utilizing the AcinetobacterPlasmidTyping database (available from https://github.com/MehradHamidian/AcinetobacterPlasmidTyping; Lam et al. 2023). p*dif* sites in the plasmids found were identified using a combination of p*dif* finder (https://github.com/mjshao06/pdifFinder) (Shao et al. 2023) and manual search as outlined by Ambrose and Hall (2024). Toxin‐antitoxin (TA) systems were determined using the TADB 3.0 database (https://bioinfo-mml.sjtu.edu.cn/TADB3/index.php) (Guan et al. 2024). IS elements were screened using ISEScan (available from https://github.com/xiezhq/ISEScan; Xie and Tang 2017) and ISfinder‐sequences database through the Prokka —*protein* option (available from https://github.com/thanhleviet/Isfinder-sequences; Siguier 2006), whereas the *comM* RI hotspot (Hamidian and Hall 2017) was determined through local BLAST.

### 2.5 Pangenome and Phylogenetic Analysis

The pangenomes of the nine Orang Asli *A. baumannii* isolates were compared with other community *A. baumannii* genomes using the Anvi'o platform (available from https://github.com/merenlab/anvio; Delmont and Eren 2018; Eren et al. 2021). Through the use of the *anvi‐pan‐genome* program, the pangenomes were determined and then visualized through *anvi‐display‐pan*, which links to the Anvi'o server. Core genome phylogenetic analysis of *A. baumannii* genomes was performed using Roary (available from https://sanger-pathogens.github.io/Roary/; Page et al. 2015) and VeryFastTree with the GTR+CAT model, which combines the General Time Reversible (GTR) nucleotide substitution model with a Constant Rate Across Sites (CAT) approximation (Piñeiro et al. 2020; Piñeiro and Pichel 2024), and visualized with iTOL v7 (Letunic and Bork 2024).

## 3 Results and Discussions

### 3.1 Preliminary Genomic Analysis of *A. baumannii* From the Orang Asli

A total of thirteen presumptive *Acinetobacter* spp. isolates were obtained from the Orang Asli carriage studies at Kampung Sungai Pergam (*n* = 3) and Kampung Berua (*n* = 10). These were from the nasopharyngeal swabs of a total of 130 participants (of which 68 were from Kampung Sungai Pergam and 62 from Kampung Berua) (Cleary et al. 2021). Whole genome sequencing was performed on all thirteen isolates, out of which nine were shown to be *A. baumannii* (Table 1). The remaining four isolates were determined to be *A. nosocomialis*.

**Table 1:** General information on the source and the genome characteristics of the *Acinetobacter baumannii* (*n* = 9) isolates from the upper respiratory tract of the Orang Asli in this study.

*Acinetobacter* isolate	19053	19055	19056	19058	19060	19061	19062	19063	19064
Location/source^a^	KSP/N	KSP/NP	KB/N	KB/N	KB/NP	KB/N	KB/NP	KB/N	KB/N
Species identification	*A. baumannii*	*A. baumannii*	*A. baumannii*	*A. baumannii*	*A. baumannii*	*A. baumannii*	*A. baumannii*	*A. baumannii*	*A. baumannii*
Accession no.	JBNPBH 000000000	JBNPBI 000000000	JBNPBJ 000000000	JBNPBK 000000000	JBNPBL 000000000	JBNPBM 000000000	JBNPBN 000000000	JBNPBO 000000000	JBNPBP 000000000
Closest neighbor (fastANI)	*A. baumannii* SAMEA 104305267 (98.1849%)	*A. baumannii* 5577 STDY7716391 (99.8833%)	*A. baumannii* 5577 STDY7716201 (97.9228%)	*A. baumannii* 5577 STDY7715392 (98.7137%)	*A. baumannii* SAMEA 104305309 (98.8467%)	*A. baumannii* SAMEA 104305313 (98.0632%)	*A. baumannii* SAMN 03174920 (98.6532%)	*A. baumannii* SAMN 03174917 (97.9238%)	*A. baumannii* SAMEA 104305269 (99.8239%)
Total genome size (bp)	3,821,374	3,697,509	3,748,932	3,904,855	3,767,717	3,865,738	3,844,544	3,828,269	3,664,686
No. of contigs	62	166	32	44	126	35	149	47	47
No. of plasmids	2	0	1	1	1	1	2	1	2
GC content (%)	38.91	39.04	38.97	38.87	38.95	38.75	38.99	38.81	38.78
N_50_	478,783	67,766	458,370	254,121	77,219	477,098	73,853	369,580	195,600
N_90_	68,035	15,343	166,877	88,568	17,967	165,195	17,420	116,120	67,830
L_50_	3	17	3	6	16	2	14	3	7
L_90_	11	61	8	18	49	8	53	10	20
No. of coding sequences	3579	3428	3497	3661	3582	3573	3674	3576	3490
MLST_Oxf_	ST3542*	ST3543*	ST3415	ST3541*	ST871	ST3544*	ST3545*	ST3540*	ST585
MLST_Pas_	ST2832*	ST2114	ST2522	ST2834*	ST2700	ST470	ST2635	ST2833*	ST10
K loci	KL121	KL96	KL170^#^	KL202^#^	KL112	KL172^#^	KL81	KL183^#^	KL108
OC loci	OCL2	OCL13	OCL2	OCL4	OCL6	OCL4	OCL6	OCL2	OCL2

*Note:* MLST_Oxf_ and MLST_Pas_ represent the Oxford and Pasteur schemes available in PubMLST, respectively. ST represents sequence type, and those marked with an asterisk (*) are new STs that were identified in this study; KL represents K loci with those labeled with ^#^ representing K loci that has not been reported; OCL represents lipo‐oligosaccharide OC loci.

^a^ Abbreviations used for location/source: GC, Global Clone; KB, Kampung Berua; KSP, Kampung Sungai Pergam; MLST, multilocus sequence typing; N, nasal swab; NP, nasopharyngeal swab; OC, outer core.

Genome assemblies of the nine *A. baumannii* Orang Asli isolates showed total genome sizes that ranged from ~3.7 to 3.9 Mbp (Table 1). ANI of the genomes revealed > 97% nucleotide identities to *A. baumannii* genomes, including those from Thailand (SAMEA104305267, SAMEA104305309, SAMEA104305313, and SAMEA104305269), Vietnam (5577STDY7716391, 5577STDY7716201, and 5577STDY7716392), and Malaysia (SAMN03174920 and SAMN03174917), as listed in Table 1. In silico epidemiological typing of the assembled genomes, which includes traditional MLST and surface polysaccharide loci typing, showed that each genome is distinct with its own ST, KL, and OCL types. MLST profiles based on the Oxford scheme revealed six new STs, with only *A. baumannii* 19056, 19060, and 19064 that were identified as preexisting ST3415_Oxf_, ST871_Oxf_, and ST585_Oxf_, respectively (Table 1). The new Oxford STs were submitted and assigned by the PubMLST curators as ST3540_Oxf_, ST3541_Oxf_, ST3542_Oxf_, ST3543_Oxf_, ST3543_Oxf_, ST3544_Oxf_, and ST3545_Oxf_ (Table 1). Additionally, the Pasteur MLST scheme identified three novel STs from the nine *A. baumannii* genomes, and these were assigned as ST2832_Pas_, ST2833_Pas_, and ST2844_Pas_. The six remaining genomes each belonged to different preexisting Pasteur STs (Table 1). The identification of new STs suggests the possible emergence of genetically distinct lineages within the Orang Asli community. The *A. baumannii* isolates with the new STs, although currently mostly susceptible, are capable of acquiring new resistance and virulence genes. Their presence in a community setting is noteworthy as they could represent a new lineage with the potential to spread and evolve, similar to how the hospital‐associated GCs, such as GC2, have emerged (Hamidian and Nigro 2019).

*A. baumannii* surface polysaccharide loci typing based on the KL, which were responsible for the production of acinetamic acid (Lam et al. 2022), showed that each of the nine Orang Asli *A. baumannii* genomes belonged to distinct KL types (Table 1). Notably, four novel capsule types (i.e., KL170, KL202, KL172, and KL183) were detected, indicating previously unreported characteristics and underscoring the uniqueness of these Orang Asli *A. baumannii* isolates. In contrast, analysis of the outer core lipo‐oligosaccharide loci (OCL) showed OCL2 (*n* = 4) to be the most common type, followed by OCL4 (*n* = 2) and OCL6 (*n* = 2) (Table 1). Nevertheless, *A. baumannii* 19055 was identified as the lesser‐studied OCL13 type, which was originally described in *A. baumannii* strains associated with community‐acquired pneumonia in the Northern Territories, Australia (Meumann et al. 2019). The *A. baumannii* capsule plays a crucial role in its ability to cause disease, primarily by protecting the bacterium from the host immune system and desiccation (Rakovitsky et al. 2021; Talyansky et al. 2021). Novel capsule types could therefore have a significant impact on the bacteria's survival and virulence.

### 3.2 Genotypic AMR Observation Revealed Nonmainstream β‐Lactamases

The presence of genes encoding β‐lactamases (*bla*) has become the hallmark of *A. baumannii*, not only as key determinants of carbapenem resistance (Shi et al. 2024), but also through the intrinsic *bla*_OXA‐51‐like_ genes, which have been adopted as one of the typing methods for *A. baumannii* over the past two decades (Shelenkov et al. 2023). The recent categorization of GCs also incorporated various *bla* genes as characteristic traits observed in certain GCs, for example, members of the GC8 lineage commonly harbor both *bla*_OXA‐23_ and *bla*_OXA‐68_ (Shelenkov et al. 2023). Although *bla* genes, such as *bla*_OXA‐23_, *bla*_OXA‐51_, and *bla*_OXA‐66_, are widespread among *A. baumannii* globally (Li et al. 2023; Shelenkov et al. 2023; Shi et al. 2024), a different spectrum of *bla* genes was found from the Orang Asli *A. baumannii* in this study (Table 2).

**Table 2:** Distribution of antimicrobial resistance determinants across the Malaysian Orang Asli *Acinetobacter baumannii* genomes (*n* = 9) and their corresponding phenotypic resistance profiles.

Antimicrobial resistance genes/phenotype	*A. baumannii* isolate								
	19053	19055	19056	19058	19060	19061	19062	19063	19064
Beta‐lactams: Resistance gene(s) Meropenem (10 µg)^a^	*bla*_ADC‐25_, *bla*_OXA‐377_	*bla*_ADC‐279_, *bla*_OXA‐120_	*bla*_ADC‐169_ variant, *bla*_OXA‐555_	*bla*_ADC‐312_ variant, *bla*_OXA‐51_	*bla*_ADC‐238_, *bla*_OXA‐510_	*bla*_ADC‐25_, *bla*_OXA‐51_	*bla*_ADC‐99_, *bla*_OXA‐98_	*bla*_ADC‐158_ variant, *bla*_OXA‐424_	*bla*_ADC‐76_, *bla*_OXA‐68_
	22.3 (S)	26.6 (S)	24.2 (S)	29.1 (S)	28.6 (S)	25.7 (S)	21.0 (S)	27.0 (S)	24.6 (S)
Aminoglycoside resistance gene(s)	*ant(3″)‐IIa*	*ant(3″)‐IIa*	—	—	*ant(3″)‐IIa*	—	*ant(3″)‐IIa*	—	*ant(3″)‐IIa*
Tetracyclines: Resistance gene(s) Tetracycline^b^ Doxycycline^b^	*tet(39)*	—	—	—	—	—	*tet(39)*	—	—
	64.0 (R)	12.0 (S)	4.0 (S)	6.0 (S)	6.0 (S)	4.0 (S)	128.0 (R)	4.0 (S)	6.0 (S)
	4.0 (S)	4.0 (S)	4.0 (S)	4.0 (S)	4.0 (S)	4.0 (S)	4.0 (S)	4.0 (S)	4.0 (S)
Sulfonamide resistance genes(s)	—	—	—	—	—	—	*sul2*	—	—
Fluroquinolones: Mutation(s) in quinolone resistance‐determining regions of *gyrA* and *parC* Ciprofloxacin (5 µg)^a^	—	—	—	—	—	—	—	—	—
	23.5 (S)	25.2 (S)	24.7 (S)	28.3 (S)	28.0 (S)	28.0 (S)	21.7 (S)	26.8 (S)	24.8 (S)
Efflux pumps	*amvA* *abeS, abeM, adeA, adeF, adeG, adeH, adeI, adeJ, adeK, adeL, adeN*	*amvA* *abeS, abeM, adeF, adeG, adeH, adeI, adeJ, adeK, adeL, adeN, adeS, adeR*	*amvA* *abeS, abeM, adeA, adeB, adeF, adeG, adeH, adeI, adeJ, adeK, adeL, adeN, adeS, adeR*	*amvA* *abeS, abeM, adeA, adeB, adeF, adeG, adeH, adeI, adeJ, adeK, adeL, adeN, adeS, adeR*	*amvA* *abeS, abeM, adeA, adeB, adeF, adeG, adeH, adeI, adeJ, adeK, adeL, adeN, adeS, adeR*	*amvA* *abeS, abeM, adeA, adeB, adeF, adeG, adeH, adeI, adeJ, adeK, adeL, adeN, adeS, adeR*	*amvA* *abeS, abeM, adeF, adeG, adeH, adeI, adeJ, adeK, adeL, adeN*	*amvA* *abeS, abeM, adeA, adeB, adeC, adeF, adeG, adeH, adeI, adeJ, adeK, adeL, adeN*	*amvA* *abeS, abeM, adeB, adeC, adeF, adeG, adeH, adeI, adeJ, adeK, adeL, adeN*

a Values indicate zone of inhibition (in mm) of respective antibiotic discs with interpretations of resistance (R) or susceptibility (S) following EUCAST (2024) breakpoints.

^b^ Values indicate minimum inhibitory concentrations (in µg/mL) measured using MIC *E* test strips with interpretations of resistance (R) or susceptibility (S) following EUCAST (2024) breakpoints.

The *bla*_OXA‐51_ family (or *bla*_OXA‐51‐like_) is comprised of the well‐characterized *bla*_OXA‐51_ gene and its numerous variants, such as *bla*_OXA‐64_, *bla*_OXA‐66_, and others (Li et al. 2023). In this study, several variants were detected in the Orang Asli *A. baumannii* genomes, including *bla*_OXA‐68_, *bla*_OXA‐98_, *bla*_OXA‐120_, *bla*_OXA‐377_, *bla*_OXA‐424_, *bla*_OXA‐510_, and *bla*_OXA‐555_. Only two genomes (i.e., *A. baumannii* 19058 and 19061) carried *bla*_OXA‐51_ itself, while the remaining seven harbored distinct variants of the *bla*_OXA‐51_ family (Table 2). The spectrum of genes of the *bla*_OXA‐51_ family aligns with the findings of Muzahid et al. (2023), who reported that community‐derived *A. baumannii* isolates from Segamat, Peninsular Malaysia, also carried a wide range of *bla*_OXA‐51_ variants. In the Segamat *A. baumannii* isolates, *bla*_OXA‐120_ was the most prevalent variant, followed by *bla*_OXA‐441_, *bla*_OXA‐510_, *bla*_OXA‐69_, *bla*_OXA‐98_, and *bla*_OXA‐412_. None of these variants were found among the *A. baumannii* isolates from the Orang Asli community.

However, the presence of the intrinsic *bla*_OXA‐51_ family of genes is not considered a definite marker for carbapenem resistance in *A. baumannii* due to the low affinities of the OXA‐51 family of β‐lactamases to meropenem and imipenem, as well as the very low expression levels of the *bla*_OXA‐51‐like_ genes. In certain cases, the presence of IS*Aba1* directly upstream of the *bla*_OXA‐51‐like_ gene provides a strong promoter which increases its expression level, leading to carbapenem resistance but this also depends on the *bla*_OXA‐51_ variant that is being overexpressed (Nigro and Hall 2018). All nine Orang Asli *A. baumannii* isolates were phenotypically carbapenem susceptible, and none of their genomes contained IS*Aba1* (or related elements) upstream of the *bla*_OXA‐51‐like_ genes. None of the nine Orang Asli *A. baumannii* isolates also harbored acquired *bla*_OXA_‐encoded carbapenemase genes, such as *bla*_OXA‐23_, *bla*_OXA‐24_, and *bla*_OXA‐58_, which have been directly implicated in carbapenem resistance, particularly in clinical *A. baumannii* isolates (Hamidian and Nigro 2019). This was similarly reported by Muzahid et al. (2023), where the acquired carbapenemase gene *bla*_OXA‐23_ was only identified in their Segamat hospital isolates, which are carbapenem resistant. Likewise, our recent study of a 10‐year collection of *A. baumannii* from the main tertiary hospital in Terengganu also revealed the predominance of the *bla*_OXA‐23_ gene among the carbapenem‐resistant isolates (Din et al. 2025).

Another class of intrinsic β‐lactamase found in *A. baumannii* genomes is the AmpC cephalosporinase variants, which are designated *Acinetobacter*‐derived cephalosporinases (ADCs). Overproduction of ADCs resulting from insertion of IS*Aba1* or similar IS elements upstream of the *bla*_ADC_ gene has been shown to be responsible for the development of resistance toward extended‐spectrum cephalosporins and, in some cases, carbapenems (Tian et al. 2011; Bhattacharya et al. 2014; Shi et al. 2024). Seven different variants of ADCs were detected from the genomes of the nine Orang Asli *A. baumannii* isolates (Table 2), and, in all cases, IS*Aba1* or similar elements were absent upstream of the encoding gene, suggesting that these genes were either not expressed or were expressed at low levels in their hosts. ADC‐25 was identified in two of the Orang Asli *A. baumannii* isolates (i.e., 19053 and 19061; Table 2), and this variant was found to be the seventh most prevalent ADC variant among *A. baumannii* isolates globally (Mack et al. 2025). Four of the eight ADC variants identified here (i.e., ADC‐99 in *A. baumannii* 19062, ADC‐238 in 19060, ADC‐279 in 19055, and ADC‐312 in 19058) were listed by Mack et al. (2025) as variants that were rarely found in *A. baumannii*. By comparison, the *A. baumannii* isolates from the Segamat community also presented a different spectrum of *bla*_ADC_ genes, where they mainly harbored *bla*_ADC‐154_ and *bla*_ADC‐156_ (Muzahid et al. 2023), but both variants were absent in our Orang Asli isolates. However, *bla*_ADC‐238_, which was found in *A. baumannii* C‐65 from the Segamat community, was also found in *A. baumannii* 19060 from our Orang Asli collection. The ADC‐238 variant was listed as a less‐frequently encountered variant (Mack et al. 2025), and its singular presence in both these community‐based studies supports this finding. Intriguingly, hospital isolates from Segamat (Muzahid et al. 2023) and Terengganu (Din et al. 2025) showed a uniform pattern of *bla*_ADC‐73_ being the most prevalent, agreeing with the analysis presented by Mack et al. (2025), which revealed *bla*_ADC‐73_ as the most prevalent ADC variant in *A. baumannii* isolates globally, with the exception of isolates from North America.

Hence, in terms of the class C (ADC) and class D (OXA) β‐lactamases, the intrinsic variants harbored by the *A. baumannii* community isolates showed diversity with little in common, although both studies (i.e., this study and the Segamat study) were in Peninsular Malaysia. Even between communities, the AMR profiles vary (Meumann et al. 2019; Muzahid et al. 2023), and thus, there is more to be learned about *A. baumannii* from the Orang Asli community, which is further elaborated in the following sections.

### 3.3 Other Resistance Genes in the Orang Asli Community *A. baumannii* Isolates

Two out of the nine Orang Asli *A. baumannii* isolates (i.e., 19053 and 19062) were found to harbor the *tet(39)* tetracycline resistance gene (Table 2), which encodes a tetracycline efflux pump of the major facilitator superfamily (Agersø and Guardabassi 2005). This differs from the *A. baumannii* community isolates from Segamat, in which no tetracycline resistance genes were detected. Both *A. baumannii* 19053 and 19062 were phenotypically tetracycline resistant (with MIC values of 64 and 128 µg/mL, respectively) but doxycycline susceptible (both with MIC values of 4 µg/mL). In contrast, Malaysian *A. baumannii* hospital isolates predominantly carried the *tet(B)* gene (*n* = 60/126 from Hospital Sultanah Nur Zahirah (HSNZ) (Din et al. 2025); *n* = 12/15 from Segamat Hospital (Muzahid et al. 2023)), with only one isolate from HSNZ harboring *tet(A)* and 10/126 carrying *tet(39)* (Din et al. 2025). All *A. baumannii* hospital isolates harboring the *tet(39)* and *tet(A)* genes were tetracycline resistant and minocycline susceptible, whereas those that harbored the *tet(B)* gene were mostly resistant to tetracycline but showed intermediate susceptibility to minocycline (Din et al. 2025). Meumann et al. (2019) reported two *A. baumannii* isolates from community‐onset pneumonia in Australia, which harbored both the *tet(B)* and *tet(39)* genes, but phenotypic susceptibility testing for tetracyclines was not performed in their study. The two tetracycline‐resistant Orang Asli *A. baumannii* isolates, 19053 and 19062, harbored the *tet(39)* gene on plasmids, which will be elaborated in a later section.

Resistance to aminoglycosides in *A. baumannii* is mainly mediated by the possession of genes encoding aminoglycoside acetyltransferase (*aac*), nucleotidyltransferase (*ant*), and/or phosphotransferase (*aph*) (Shi et al. 2024). Five out of the nine *A. baumannii* Orang Asli isolates carried the *ant(3″)‐IIa* gene (Table 2), whereas Muzahid et al. (2023) reported the presence of this gene in all 12 of their *A. baumannii* strains that were isolates from the community in the town of Segamat. Nevertheless, only two of the 12 Segamat community isolates showed resistance to amikacin, and all were gentamicin susceptible, suggesting that some of these aminoglycoside resistance genes were either not expressed or expressed at a very low level. Phenotypic resistance to aminoglycosides was, however, not tested for the Orang Asli *A. baumannii* isolates in this study.

Sulfonamide resistance in *A. baumannii* is usually mediated by *sul1* and/or *sul2* genes (Sköld 2001), with *sul2* predominantly reported from Southeast Asian countries and the Asia‐Pacific region (Bian et al. 2021; Brito et al. 2022; Din et al. 2025). Only one Orang Asli isolate, *A. baumannii* 19062, was found to harbor the *sul2* gene (Table 2), which was absent in the Segamat community isolates (Muzahid et al. 2023). In contrast, nearly 50% (61/126) of the *A. baumannii* hospital isolates from HSNZ, Terengganu, harbored the *sul2* gene (Din et al. 2025), whereas in Hospital Segamat, the gene was identified in 2/15 of the *A. baumannii* isolates. The significance of the carriage of the *sul2* gene in the solitary Orang Asli *A. baumannii* isolate is currently unknown, but *sul2* is known to be present on mobile elements, such as plasmids and transposons (Jeon et al. 2023). Plasmid analysis appeared to rule out the carriage of *sul2* in either of the two plasmids found in *A. baumannii* 19062 (see subsequent Section 3.7), but this does not rule out its location on other mobile elements such as transposons or genomic islands in the chromosome.

### 3.4 The Virulome of *A. baumannii* From the Orang Asli Community

**Figure 1:**
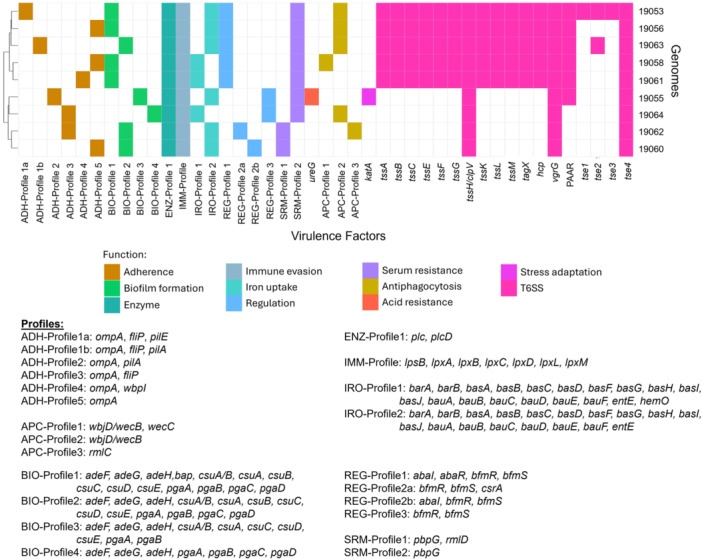
Profiles of various virulence factors (VFs) identified from the Orang Asli *Acinetobacter baumannii* genomes (*n* = 9). The profiles were abbreviated according to their biological functions: adhesion (ADH), antiphagocytosis (APC), biofilm formation (BIO), phospholipase enzyme (ENZ), immune evasion (IMM), iron uptake (IRO), regulation (REG), and serum resistance (SRM); singleton genes were labeled as is.

The isolation of *A. baumannii* from diverse sources, including clinical settings, soil, and wastewater, highlights its ability to persist across various environmental niches, facilitating its widespread dissemination, colonization, and pathogenicity (Harding et al. 2018). This persistence, evidenced by stress resistance and biofilm formation, is likely supported by the acquisition and/or inheritance of multiple virulence factors (VFs). In this study, the majority of identified VFs are associated with adhesion, biofilm formation, and quorum‐sensing regulation (Figure 1). These findings are in agreement with Muzahid et al. (2023), who reported similar virulome profiles in *A. baumannii* isolates from the Segamat community, including genes related to adhesion (e.g., *ompA*, *fliP*, *pilA*, and *pilE*), biofilm formation (e.g., *adeFGH*, *bap*, *csuA/B*, *csuABCDE*, and *pgaABCD*), and quorum sensing (e.g., *abaI* and *abaR*) (Choi et al. 2009; Lannan et al. 2016; Ahmad et al. 2023). However, slight variations in VF combinations were observed between these two Malaysian community studies. Notably, the biofilm gene combination we termed BIO‐Profile 1 (Figure 1) was dominant in our isolates but was absent in those from the Segamat community. Despite these differences, the presence of numerous shared VFs underscores their potential role in supporting the persistence of the *A. baumannii* isolates in their niche and their capacity to cause infection.

Survival of *A. baumannii* in harsh environmental conditions requires mechanisms for the acquisition of micronutrients, such as iron scavenging through the production of acinetobactin in iron‐limiting environments (Lannan et al. 2016; Harding et al. 2018). The presence of iron uptake genes is thus a signature VF in *A. baumannii* and the combination of iron acquisition genes designated IRO‐Profile2 (Figure 1) was also observed in the Segamat community isolates along with isolates from the Australian Northern Territory community (Meumann et al. 2019; Muzahid et al. 2023).

All nine *A. baumannii* isolates displayed the full complement of the *lps‐lpx* genes (designated IMM‐Profile; Figure 1), which are tagged as VFs that function in immune evasion. Deficiency in *lpxC* has been shown to cause the loss of the LPS layer in *A. baumannii*, leading to the development of colistin resistance (Kamoshida et al. 2020). The full suite of the *lps‐lpx* genes was found in the Segamat *A. baumannii* isolates, and this included hospital isolates that were identified as resistant to colistin (*n* = 2) and polymyxin B (*n* = 4); nevertheless, a more detailed analysis of possible mechanisms for polymyxin resistance, including mutations in the *lps‐lpx* genes, was not presented (Muzahid et al. 2023).

The Type‐6 Secretion System (T6SS) is utilized by *A. baumannii* to release toxic effector proteins into the neighboring environment, offering a competitive advantage to the pathogen in multispecies environments (Carruthers et al. 2013) and also allowing *A. baumannii* to spread, invade, and resist host immune responses (Shadan et al. 2023). The T6SS main cluster (T6MC), which encompasses the *tssA*, *tssB*, *tssC*, *tssD*/*hcp*, *tssE*, *tssF*, *tssG*, *tssH*/*clpV*, *tssK*, *tssL*, *tagX*, *vgrG*, and PAAR genes (Fitzsimons et al. 2018; Lewis et al. 2019), was identified in five of the nine Orang Asli *A. baumannii* isolates (Figure 1). These genes are responsible for T6SS apparatus assembly, whereby TssA functions as the priming protein (also known as the cap), TssBC forms the sheath, TssD/Hcp the secretion tube with VgrG and PAAR proteins as the spike. The spike (i.e., VgrG and PAAR) teams with the wedge (i.e., TssK and TssEFG) to form the baseplate (Fitzsimons et al. 2018; Marazzato et al. 2022). The structure was then supported by the membrane complex formed by TssJ, TssM, and TssL proteins connecting between inner and outer membranes (Fitzsimons et al. 2018; Marazzato et al. 2022). The T6SS found in the five *A. baumannii* genomes (Figure 1) was further identified as T6SS‐1A (i.e., 19053, 19056, and 19063) and T6SS‐1B (i.e., 19058 and 19061), according to the classification of Repizo et al. (2019). These five *A. baumannii* isolates also encode the Tse4 effector (Figure 1), which function as an amidase (Lewis et al. 2019; Repizo et al. 2019). Other effectors were also present in the Orang Asli isolates, with *A. baumannii* 19063 and 19053 encoding an additional Tse2 (predicted to function as a DNase), while *A. baumannii* 19053 also encodes additional Tse1 (predicted lipase producer), and Tse3 (effector of unknown function) (Lewis et al. 2019; Repizo et al. 2019). Conversely, only one of the Segamat community isolates, *A. baumannii* C‐98, harbored the complete T6SS, whereas the hospital isolates contained the full suite of T6SS genes (Muzahid et al. 2023). The majority of the *A. baumannii* hospital isolates from Terengganu also harbored the full T6MC (*n* = 94/126; or 74.6%) (Din et al. 2025). This suggests that the Orang Asli *A. baumannii* isolates may be better adapted to survival in a multispecies environment with also the capacity to invade and colonize their host, should the opportunity arise; however, such possibilities would require further experimental validation.

### 3.5 The Orang Asli *A. baumannii* Isolates Were Genetically Diverse

**Figure 2:**
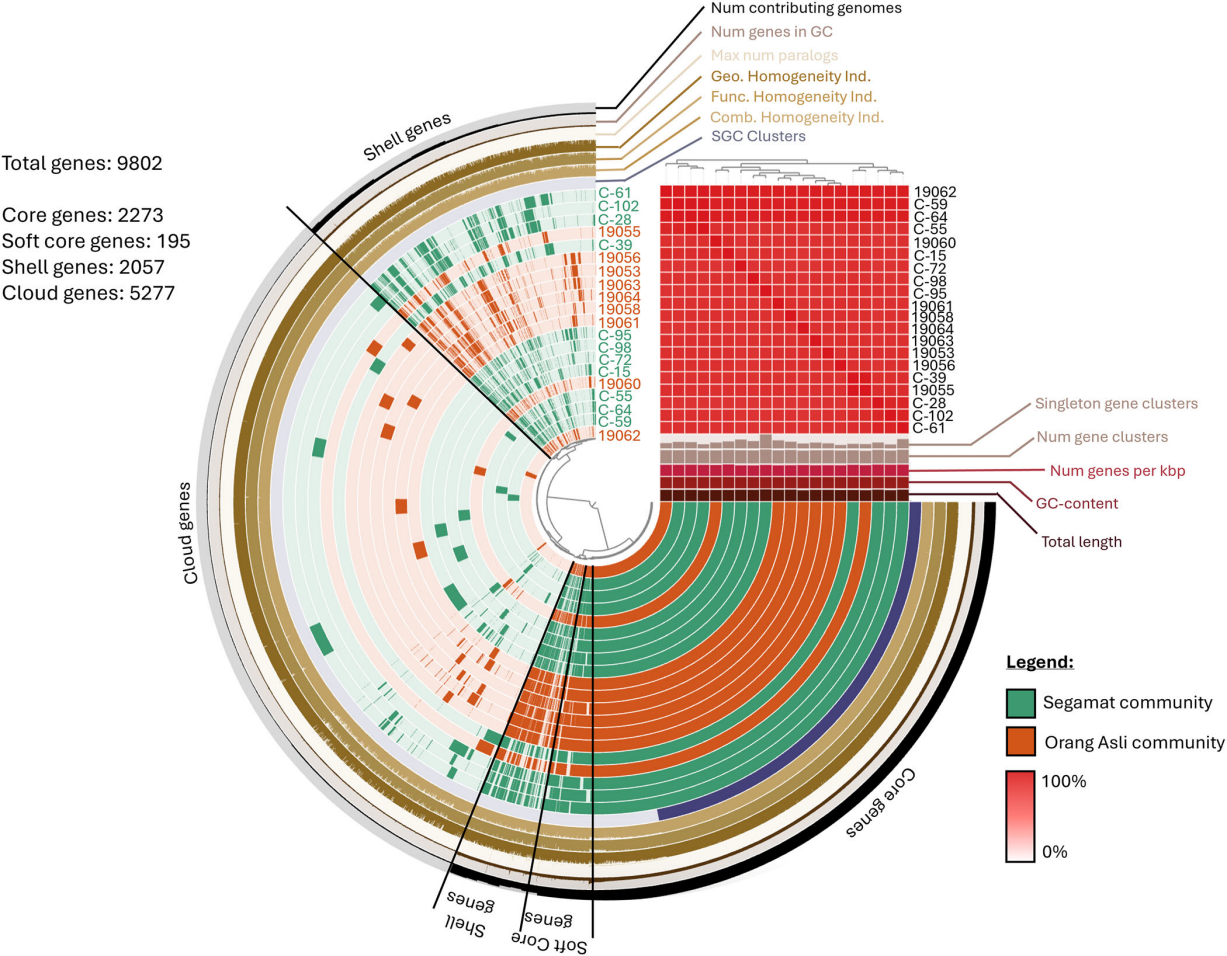
Pangenome analysis of community *Acinetobacter baumannii* isolates from Malaysia that are currently available in the databases (*n* = 20). The orange‐colored tracks represent the Orang Asli community *A. baumannii* from this study (*n* = 9), whereas the green‐colored tracks represent the Segamat community strains (*n* = 11; isolates with the prefix “C”) that were previously published (Muzahid et al. 2023). Heatmap on the top right corner presents the average nucleotide identity (ANI) of *A. baumannii* from both communities, with all of them having > 97% identity. GC, Global Clone.

**Figure 3:**
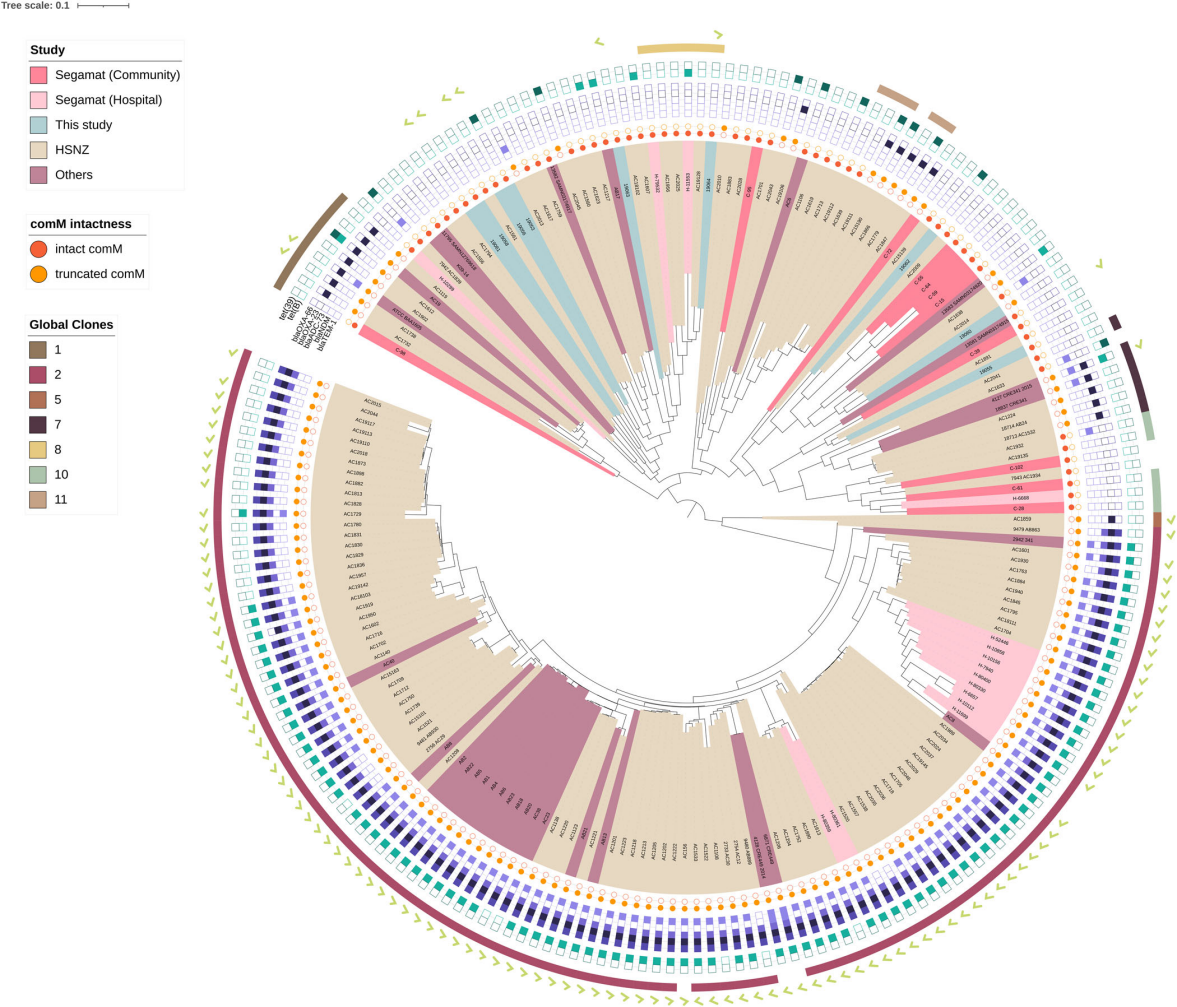
Midpoint‐rooted maximum‐likelihood phylogenetic tree of all Malaysian *Acinetobacter baumannii* genomes that are published (i.e., the current Orang Asli isolates, the Segamat hospital and community isolates described by Muzahid et al. 2023, and the 10‐year HSNZ isolates reported by Din et al. 2025), along with *A. baumannii* genomes originating from Malaysia that are found in PubMLST (as of January 30, 2025). Also shown are their categorization into the various Global Clone (GC) lineages, the presence/absence of predominant carbapenemase (purple boxes) and tetracycline resistance genes (green boxes), the intactness of the *comM* gene (orange circles), and the presence of plasmids of the Rep_3 family (light green tick marks). HSNZ, Hospital Sultanah Nur Zahirah.

Community isolates of *A. baumannii* represent a pool of unexplored genomes when compared with the more well‐studied clinical isolates. Therefore, many *A. baumannii* isolates presented in community studies belonged to novel STs that have yet to be classified into any clonal complexes. We pooled together the *A. baumannii* genomes obtained from the Orang Asli in this study, along with the genomes obtained from fecal samples of the community in the town of Segamat (Muzahid et al. 2023), for pangenome analysis. The analysis showed genetic diversity among these community isolates from the 5277 cloud genes identified (53.84%) when compared with the 2273 core genes (23.19%) (Figure 2). The lower ratio of core genes indicated low homogeneity between the *A. baumannii* community isolates, highlighting the uniqueness of the bacterium in each population. Even within the Segamat population itself, the higher diversity of the community *A. baumannii* isolates was apparent, as compared with the hospital isolates from the same town (Muzahid et al. 2023). Of interest, there did not seem to be any apparent geographical clustering between the two populations (Figure 2), and this was evident when examining the core genome phylogenetic tree that was generated using both community and clinical isolates of *A. baumannii* from Malaysia (Figure 3). Segamat is a township in the state of Johor and is approximately 390 km south of the state of Terengganu, where the sampling was carried out in the Orang Asli rural settlements.

A maximum‐likelihood phylogenetic tree was generated from the core genome alignment of 199 Malaysian *A. baumannii* genomes (Figure 3), and these included the genomes from this study, the Segamat study (both community and hospital isolates) (Muzahid et al. 2023), 126 genomes from a 10‐year collection of isolates from HSNZ, the main tertiary hospital in Terengganu (Din et al. 2025), and other clinical isolates of *A. baumannii* obtained from the PubMLST database (refer to Supporting Information Appendix 1 for the list of genomes). Hospital isolates of *A. baumannii* were predominantly ST2_Pas_, which were categorized under the GC2 lineage, and this is clearly evident in the phylogenetic tree where they are clustered in a distinct clade (Figure 3). GC2 is the predominant *A. baumannii* lineage globally (Nigro and Hall 2018), but the basis of this predominance is that the overwhelming majority of sequenced isolates were from hospitals (Shelenkov et al. 2023). As mentioned earlier, the Orang Asli *A. baumannii* genomes were scattered throughout the phylogenetic tree, as were the Segamat community isolates, whereas most of the Segamat hospital isolates were clustered together in the GC2 clade, much like the HSNZ isolates. Interestingly, one Orang Asli isolate, *A. baumannii* 19064 (ST10_Pas_; ST585_Oxf_), was found to be a member of the GC8 lineage. Similarly, Muzahid et al. (2023) had reported that one of their community isolates from Segamat was identified as a member of the GC1 lineage. Apart from these exceptional cases, none of the community *A. baumannii* isolates belonged to any GC clusters. Additionally, the Orang Asli *A. baumannii* was also distinct from the Segamat community isolates, with only *A. baumannii* 19062 distantly grouped with C‐72 (Figure 3). Although the majority of the Orang Asli isolates did not belong to any of the major GCs, we observed that they were interleaved with a few non‐GC clinical isolates from HSNZ in neighboring branches (Figure 3). It is possible that these community *A. baumannii* isolates were able to cause infections whenever the opportunity arises (i.e., potential pathogenicity), and thus, we see the relatively close genetic relationship between some of the Orang Asli isolates and the non‐GC hospital isolates. However, without specific infection data and proven clinical relevance, this remains speculative. Nevertheless, genomic data can inform future research on *A. baumannii*, particularly with broader longitudinal carriage studies, contact tracing, or case control analysis with clinical samples, and any transmission between the community and hospital isolates could be detected and proven.

Apart from the distinct clustering of various GCs (Figure 3), the Malaysian clinical *A. baumannii* isolates also presented a different catalog of dominant *bla* genes, which varies from the community *A. baumannii* resistome described earlier. The presence of *bla*_TEM‐1_ (Ambler Class A), *bla*_NDM‐1_ (Ambler Class B), *bla*_ADC‐73_ (*bla*_ADC‐1‐like_; Ambler Class C), *bla*_OXA‐23_, and *bla*_OXA‐66_ (Ambler Class D) was recorded from almost all the Malaysian GC2 genomes, and likewise for the tetracycline resistance gene *tet(B)* (Figure 3). One distinctive feature of *A. baumannii* hospital isolates is the presence of large AbaR RIs that were often inserted within the chromosomal *comM* gene (Meumann et al. 2019). The intactness of the *comM* gene was investigated for all the Malaysian *A. baumannii* genomes presented here. Not surprisingly, the *comM* gene was interrupted in all the GC2 isolates, whereas the proportion was 87.5% for GC1, 71.4% for GC7, and 66.7% for GC11 isolates (Figure 3). The non‐GC *A. baumannii* genomes have a much lower percentage of interrupted *comM*, and within the nine Orang Asli isolates, only *A. baumannii* 19060 was identified with disruption of the *comM* gene (Figure 3). Nevertheless, the genes that were inserted within *comM* in *A. baumannii* 19060 were not associated with AMR but rather those associated with metabolism, regulatory genes, and hypothetical proteins, much like what was described for the community‐onset isolates from the Australian Northern Territory (Meumann et al. 2019).

### 3.6 IS Elements

A total of 17 ISs belonging to five families, namely, IS*3*, IS*5*, IS*91*, IS*630*, and IS*L3*, were identified from the Orang Asli *A. baumannii* genomes (Supporting Information Appendix 2). IS*Aba43* of the IS*L3* family (Cameranesi et al. 2020) was found in all nine *A. baumannii* genomes, with at least one copy of the IS in each genome (Supporting Information Appendix 2). Other IS families were found in fewer numbers with IS*Aba40*, IS*Aba57*, and IS*Aba63* of the IS*3* family found in one genome each, and the remaining IS elements identified were found only in *A. baumannii* 19062 (Supporting Information Appendix 2). There were few reports for most of these IS elements except IS*1006*, which was identified in *A. baumannii* 19062 and is well‐known for its association with plasmid‐borne AMR regions (Harmer and Hall 2021; Varani et al. 2021; Hall 2022). However, the identified copy of IS*1006* in *A. baumanii* 19062 was not found to be associated with any resistance genes.

### 3.7 Carriage of Plasmids in *A. baumannii* From the Orang Asli Community

Plasmids play an important role in the evolution of *A. baumannii*, being a major vehicle for the dissemination of antibiotic resistance genes (Lam and Hamidian 2024; Tobin et al. 2025). In a comprehensive survey of 439 mostly complete *A. baumannii* genomes, more than half (52%) contained one plasmid, 27% harbored two plasmids, while seven genomes contained 6–11 plasmids (Lam and Hamidian 2024). Plasmids of the Rep_3 family were by far, the most predominant with more than half of these plasmids harboring antibiotic resistance genes (Lam et al. 2023; Lam and Hamidian 2024; Tobin et al. 2025). Among the nine Orang Asli *A. baumannii* genomes, only one isolate, *A. baumannii* 19055, was without any detectable plasmids; three isolates (i.e., 19053, 19062, and 19064) harbored two plasmids each, while the remaining five isolates contained a plasmid each (Supporting Information Appendix 3). Eight of these plasmids were small plasmids (i.e., < 10 kb; Lean and Yeo 2017), ranging in size from 2178 to 8837 bp. Out of these eight small plasmids, only one plasmid, p19053a, belonged to the Rep_1 family, specifically the R1‐T6 type. p19053a was only 2178 bp and harbored the *rep* gene and two other hypothetical open reading frames (ORFs) (Supporting Information Appendix 4). Lam and Hamidian (2024) noted that the R1‐type plasmids are typically 2–3 kb in size and encode only the replication initiation protein along with one or two hypothetical proteins. None of these plasmids harbored AMR genes and p19053a follows the characteristics of the prototypical R1‐type plasmid.

**Figure 4:**
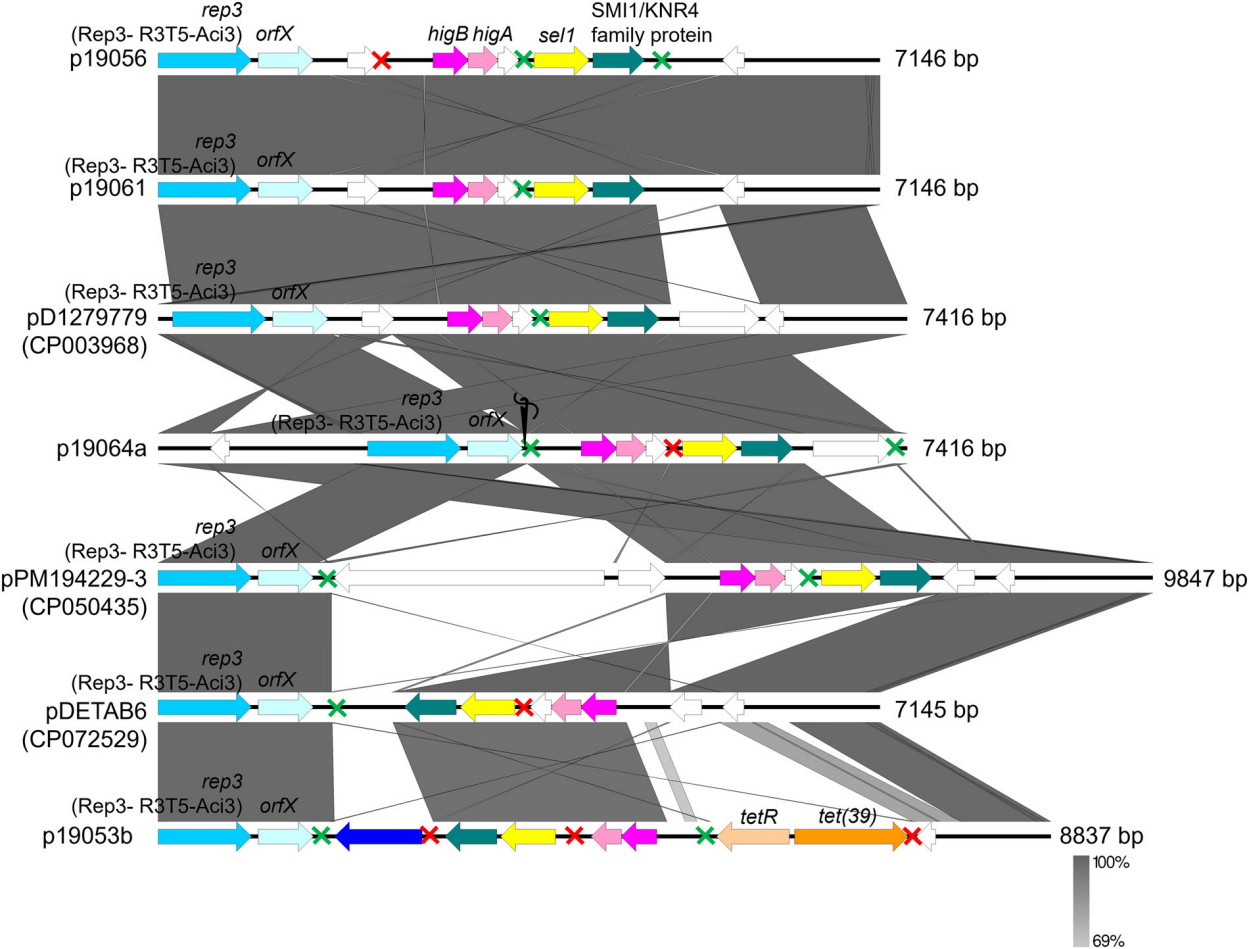
Comparisons of the R3‐T5‐type plasmids found in the Orang Asli *Acinetobacter baumannii* isolates with similar R3‐T5‐type plasmids in the database curated by Lam et al. (2023). The needle and thread icon shown for the p19064a plasmid map indicated that the plasmid was a composite that was stitched together from two separate contigs. The p*dif* sites are marked with green and red crosses representing XerC/D and XerD/C recognition sites, respectively.

The remaining seven small plasmids were of the Rep_3 family, specifically the R3‐T5 (*n* = 4), R3‐T13 (*n* = 2), and R3‐T64 (*n* = 1) types (Supporting Information Appendices 3 and 4). Two of these R3 types (i.e., R3‐T5 and R3‐T13) were reportedly among the most abundant R3 types globally, but their distributions were mainly linked to minor STs (Lam and Hamidian 2024). Plasmid p19064a from the R3‐T5 type was found in an ST10_Pas_ host (*A. baumannii* 19064), which was the only isolate from this study that belonged to one of the known GC lineages, GC8, but did not harbor antibiotic resistance genes. Among these small plasmids, only p19053b, which was also of the R3‐T5 type, harbored the *tet(39)* tetracycline resistance gene (Figure 4). In contrast, *A. baumannii* clinical isolates from Malaysia, particularly those of the GC2 lineage, prevalently harbored an 8731‐bp plasmid we initially designated pAC12a (Lean et al. 2014; Lean and Yeo 2017; Din et al. 2025) and which is identical to the pA1‐1 plasmid (accession no. CP010782) that is harbored in a GC1 isolate obtained in 1982. This plasmid was previously typed as GR2 in an older scheme (Bertini et al. 2010) but has since been typed as R3‐T1, and almost half of the members of this group were identical or nearly identical to pA1‐1 (Lam et al. 2023). This supports the long‐standing association of this plasmid with the GC1 and GC2 lineages, but isolates of other STs have also been found that harbor this plasmid, albeit at a much lower frequency (Lam et al. 2023; Din et al. 2025). A characteristic feature of this plasmid is the presence of mobile p*dif* modules containing the *sel1*, *abkBA* TA genes, and a gene encoding a TonB‐dependent receptor (Lean and Yeo 2017; Lam et al. 2023). Notably, this plasmid type was absent from all *A. baumannii* isolates obtained from the Orang Asli population and the Segamat community, suggesting a possible specific association with clinical strains.

**Figure 5:**
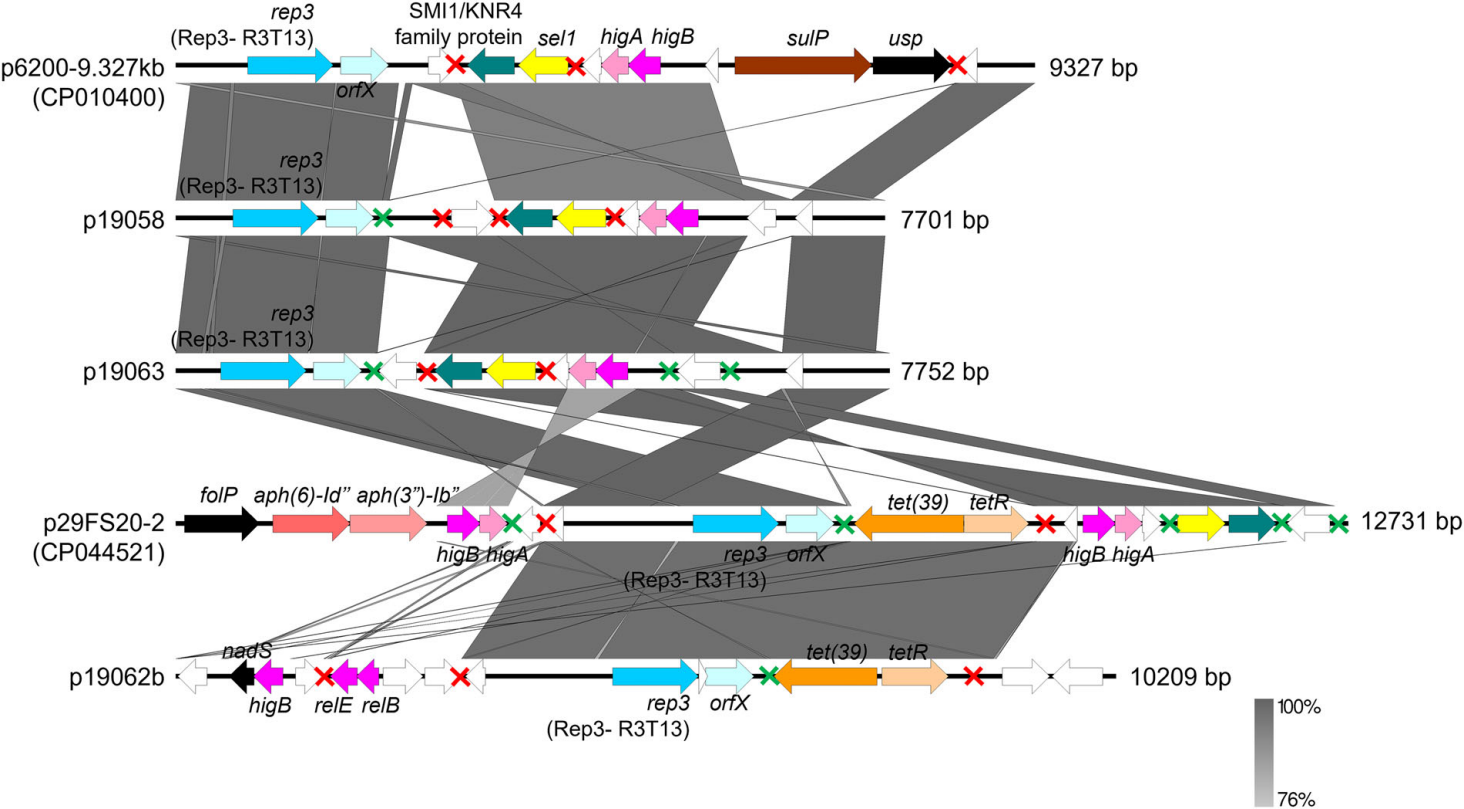
Comparative linear map of R3‐T13‐type plasmids identified from the Orang Asli *Acinetobacter baumannii* with closely related R3‐T13‐type plasmids found in the plasmid database presented by Lam et al. (2023). Red–green crosses representing p*dif* sites were labeled as indicated in the previous Figure.

Lam and Hamidian (2024) reported that almost half of the R3‐type plasmids were not associated with AMR determinants. The genetic structures of the R3‐T5 and R3‐T13‐type plasmids identified in this study (*n* = 6; Figure 4 and Supporting Information Appendix 4) showed the absence of AMR genes from five of them, except for the *tet(39)–tetR* genes in p19053b. The *tet(39)–tetR* genes are located within a mobile p*dif* module usually found in *Acinetobacter* plasmids (Blackwell and Hall 2017; Lean and Yeo 2017). A p*dif* module typically comprises one or two related genes flanked by p*dif* sites, which resemble the chromosomal *dif* site involved in site‐specific recombination. These 28 bp p*dif* sites contain binding regions for the recombinases XerC and XerD, similar to the chromosomal *dif* site near the bacterial chromosome terminus. The term p*dif* was used to differentiate these plasmid‐associated sites from chromosomal *dif* sites (Blackwell and Hall 2017; Castillo et al. 2017; Balalovski and Grainge 2020). Three other p*dif* modules were uncovered from p19053b, and these carry a nucleotide‐binding protein‐encoding gene, *higBA* TA genes, and a *sel1* gene along with a gene encoding the SMI1/KNR4‐family protein (Figure 4; Supporting Information Appendix 5). Interestingly, the gene arrangement of the *higBA* and *sel1*‐SMI1/KNR4‐family p*dif* modules was observed in almost all the R3‐T5‐type plasmids identified in this study and also extends to the R3‐T13‐type plasmids (i.e., p19058 and p19063) (Figures 4 and 5; Supporting Information Appendix 4).

Two plasmids of the R3‐T5‐type, namely, p19056 and p19061, were nearly identical, with both at 7146 bp (Figure 4). They were closely related to the pD1279779 plasmid (accession no. CP003968.1), which was slightly larger at 7416 bp with an additional hypothetical protein found downstream of the SMI1/KNR4‐family gene (Figure 4). Plasmid p19064a also appeared to be closely related to pD1279779, but the p19064a sequence was actually stitched together from two separate contigs (Figure 4), and whether there are additional genes that are lost in between these two contigs is not known. Interestingly, the host for pD1279779 was *A. baumannii* D1279779, which was isolated from a community‐acquired bacteremia patient in Northern Australia and typed as ST267_Pas_/ST942_Oxf_ (Farrugia et al. 2013). On the basis of the collection of *Acinetobacter* plasmids curated by Lam et al. (2023), the R3‐T5‐type plasmids identified in this study were related to pPM194229‐3 and pDETAB6, besides pD1279779. The p19053b plasmid, which harbored the *tet(39)–tetR* genes, is thus the sole R3‐T5‐type plasmid, which encode AMR determinants. This plasmid is more closely related to pDETAB6, which harbored two hypothetical ORFs instead of the *tet(39)–tetR* genes in p19053b (Figure 4).

AMR genes are also a rarity among the R3‐T13‐type plasmids. A search in the *Acinetobacter* plasmid repository posted by Lam et al. (2023) led to the discovery of p29FS20‐2 (accession no. CP044521.1) as the sole carrier of *tet(39)–tetR*, and the aminoglycoside resistance genes *aph(3″)‐Ib* and *aph(6)‐Id* in this plasmid type. Two of the three R3‐T13‐type plasmids identified in this study, p19058 and p19063, were not associated with AMR determinants; however, p19062b was the only R3‐T13‐type plasmid that harbored the *tet(39)–tetR* gene pair (Figure 5). Similar to p19053b, the *tet(39)–tetR* genes in p19062b were also located within a p*dif* module, a feature that was also observed in p29FS20‐2 (Figure 5). Plasmids p19058 and p19063 were more closely related to another R3‐T13‐type plasmid, p6200‐9.327 kb, but this plasmid contained additional genes encoding sulfate permease (*sulP*) and a universal stress protein (*usp*) (Figure 5). One of the interesting observations of note regarding these plasmids is the almost universal presence of the *rep_3‐orfX* backbone, the *higBA*, and *sel1‐*SMI1/KRN4 family modular arrangement of genes, covering also the R3‐T5‐type plasmids (Figures 4 and 5). The sole exception to this is plasmid p19062b, where the *sel1*‐SMI1/KRN4 p*dif* module was absent, and the putative TA module (identified as *relBE* by TADB 3.0) was distantly related to the *higBA* module usually found in the other similar plasmid‐types (Figure 5).

The other plasmids identified in the Orang Asli *A. baumannii* genomes are p19062a of the R3‐T64 type, p19060 of R3‐T26, and p19064b of R3‐T27 type (Supporting Information Appendix 4). These plasmid types are rarely encountered when compared with the R3‐T5 and R3‐T13 types (Lam et al. 2023). None of these plasmids harbor AMR determinants.

Even though only nine *A. baumannii* genomes were investigated in this study, the diversity of plasmids found reflects the diversity reported for the genus *Acinetobacter* (Lam and Hamidian 2024). Given the limited direct clinical antibiotic exposure in the studied community, it is plausible that plasmid‐mediated resistance (in particular, tetracycline resistance) is maintained and disseminated through environmental and ecological interactions. Local environmental sources (such as soil and water contaminated with antimicrobial residues) and animals (including livestock and wildlife) can act as reservoirs of resistance plasmids and provide opportunities for gene exchange between commensal, environmental, and pathogenic bacteria. Such interactions may help explain the persistence and spread of resistance determinants in these isolates despite low human antibiotic usage (Davies and Davies 2010; Aminov 2009). This is particularly true for tetracycline resistance, in which the antibiotic has been extensively used for decades in both human and veterinary medicine as well as in agriculture and aquaculture. This historical use could have created an initial selective pressure which led to the persistence of the resistance gene(s) in the bacterial population (Aminov 2009). Even if the exposure to tetracycline is now limited, the fitness cost of carrying the resistance gene might be low, which enables them to persist by being integrated into mobile genetic elements such as p*dif* modules and plasmids that are easily transmitted between bacteria. The *tet(39)* p*dif* module along with p*dif* modules harboring carbapenem and macrolide resistance genes have indeed been reported in diverse *Acinetobacter* spp. isolated from aquatic environments in South Australia (Tobin et al. 2024).

### 3.8 Perspectives, Limitations, and Suggestions for Future Studies

By conducting genomic analysis of *A. baumannii* strains from a previously understudied population, our work addresses a key WHO priority in its Global Action Plan on AMR (WHO 2015). The identification of resistance genes and their prevalence in this unique community provides new surveillance data from a region where such information is limited. This is essential for informing and strengthening national and regional AMR strategies, thereby contributing to the global surveillance network. Our findings on the presence of antimicrobial‐resistant *A. baumannii* in asymptomatic carriers from a community setting also underscore the importance of community‐level surveillance and infection prevention. WHO recognizes that AMR is not just a hospital problem (WHO 2015). For *A. baumannii*, there has been scarce data on the genomic characteristics of community‐origin isolates worldwide, resulting in the overwhelming prevalence of hospital‐origin GC2 genomes in the public databases (Lam and Hamidian 2024; Shelenkov et al. 2023; Hamidian and Nigro 2019). Our study also highlights the need for targeted public health interventions and awareness campaigns to mitigate the spread of resistant bacteria in nonclinical settings. This information is vital for developing effective community‐based strategies to limit the transmission of AMR.

A major limitation in this study is that these *A. baumannii* genomes were sequenced using the Illumina short‐read platform, which does not allow for complete genome assembly. Therefore, some of the plasmid architectures presented here should be taken with caution, as we could not ascertain if there are genes or genetic elements that are lost in the assembly of the draft genomes. This limitation is particularly relevant for plasmid p19064a, which was assembled across two separate contigs. Nevertheless, the fact that some of these plasmids have similar counterparts from clinical *A. baumannii* isolates is of concern, as they could serve as vehicles for the dissemination of AMR genes. The discovery of the *tet(39)–tetR* gene pair within a mobile p*dif* module in two distinct plasmids, p19053b and p19062b, underlines this likelihood and highlights the importance of continued genomic surveillance, particularly among the community.

Another limitation of this study is the small number of *A. baumannii* isolates that were obtained and analyzed (*n* = 9), thus necessitating future broader longitudinal studies for a better representation of the population. We also phenotypically tested a limited number of antimicrobials in this study (i.e., meropenem, ciprofloxacin, tetracycline, and doxycycline). Hence, increasing the number of antibiotics by including classes such as the aminoglycosides would be useful for a more comprehensive phenotypic resistance profile, more so as the aminoglycoside resistance gene, *ant(3″)‐IIa*, was detected in five isolates. The expression of these resistance genes can be validated by using quantitative real‐time reverse transcriptase PCR (qRT‐PCR), particularly for isolates in which the resistance gene is detected but the isolate remains phenotypically susceptible. Future studies should also focus on functional assays to determine the role of the new STs and capsule types in biofilm formation, resistance to environmental stresses, and host immune evasion. This will provide a clearer understanding of how these novel types might influence the ability of the bacteria to cause disease and resist antibiotics in clinical settings.

## 4 Conclusions

*A. baumannii* and its AMR mechanisms have long been central in the global fight against superbugs, as understanding these traits is essential for improving treatment strategies. Over the decades, numerous reports have highlighted the alarming rise in carbapenem‐resistant, MDR, and even PDR *A. baumannii* strains in clinical settings (Akeda 2021; C. H. Chen et al. 2023), suggesting that treatment practices themselves may contribute to the development of resistance (De Blasiis et al. 2024). In contrast, *A. baumannii* isolates from remote communities, such as the indigenous Orang Asli population studied here, exhibited lower levels of antibiotic resistance. However, data on community‐derived isolates remain scarce when compared with clinical isolates (Lam and Hamidian 2024).

Despite their relative antibiotic susceptibility, these community‐associated *A. baumannii* isolates still harbor a range of VFs and mobile genetic elements, including plasmids and ISs (Meumann et al. 2019; Muzahid et al. 2023), features well‐documented in hospital‐derived strains. Notably, one isolate from this study belonged to the GC8 clinical lineage, while Muzahid et al. (2023) previously identified a GC1 isolate in a more urbanized community setting in Malaysia. Moreover, the phylogenetic interleaving of these community isolates with certain non‐GC hospital strains suggests that they possess the capacity to cause infections and acquire resistance traits, akin to their clinical counterparts. The presence of shared genomic features in *A. baumannii* from a presumptive antibiotic‐naïve environment underscores the pathogen's inherent capacity for persistence and colonization, even in healthy individuals. Of concern is the detection of two *A. baumannii* isolates from this study that harbors tetracycline resistance genes in mobile p*dif* modules located in distinct plasmids with similarities to those isolated from clinical strains. This finding suggests the potential for further acquisition of resistance determinants and serves as a cautionary signal against the unregulated introduction of antibiotics into vulnerable populations, highlighting the need for a targeted public health policy that guides the use of antibiotics in these communities. Healthcare practitioners working with remote communities can use these insights to make more informed treatment decisions, adopting rapid diagnostic tests, if possible, to ensure that antibiotics are prescribed only when clinically necessary. This minimizes unnecessary antibiotic exposure, thereby potentially reducing the emergence and spread of AMR (Yau et al. 2021). Therefore, expanding genomic surveillance to include community‐derived *A. baumannii* strains, even from remote indigenous tribes, is indeed a useful endeavor. Although this is a road less taken, the knowledge obtained, particularly tracking the shifts in known and novel STs, will be invaluable for understanding the pathogen's broader epidemiological dynamics and informing future public health strategies.

## Author Contributions

**Soo Sum Lean:** conceptualization (equal), data curation (lead), formal analysis (lead), investigation (equal), methodology (lead), software (lead), visualization (lead), writing – original draft (lead), writing – review and editing (equal). **Denise E. Morris:** investigation (equal), methodology (equal), project administration (equal), resources (equal). **Rebecca Anderson:** investigation (equal), methodology (equal), project administration (equal), resources (equal). **Ahmed Ghazi Alattraqchi:** investigation (equal), methodology (equal), resources (equal), software (equal). **David W. Cleary:** conceptualization (equal), formal analysis (equal), funding acquisition (equal), resources (equal), software (equal), supervision (equal), writing – review and editing (equal). **Stuart C. Clarke:** conceptualization (equal), funding acquisition (lead), supervision (lead), resources (lead), writing – review and editing (equal). **Chew Chieng Yeo:** conceptualization (equal), formal analysis (equal), resources (equal), supervision (equal), validation (equal), writing – original draft (equal), writing – review and editing (equal).

## Ethics Statement

Ethical approval for isolates taken in Peninsular Malaysia was provided by Universiti Sultan Zainal Abidin (UniSZA) Ethics Committee: Approval No. UniSZA/C/1/UHREC/628–1(85) dated June 27, 2016, the Department of Orang Asli Affairs and Development (JAKOA): Approval No. JAKOA/PP.30.052Jld11[42], and by the University of Southampton Faculty of Medicine Ethics Committee (Submission ID 20831). Written informed consent was taken with parents/guardians providing consent for those < 18 years old.

## Consent

All authors have provided consent for publication.

## Conflicts of Interest

The authors declare no conflicts of interest.

## Supporting information

Appendix.

## Data Availability

The nine *Acinetobacter baumannii* genomes have been deposited in the National Center for Biotechnology Information (NCBI)'s Genomes database under BioProject no. PRJNA1258958.
