# Supplementary material for: The Road Less Traveled: Exploring the Genomic Characteristics and Antimicrobial Resistance Potential of Acinetobacter baumannii From the Indigenous Orang Asli Community in Peninsular Malaysia
Source: Microbiologyopen. 2025 Oct 17;14(5):e70073. doi: 10.1002/mbo3.70073 (PMC12531904; doi:10.1002/mbo3.70073)
Supplement: Supplementary file 1 — Appendix. [file MBO3-14-e70073-s001.docx]

**APPENDIX 1:** List of *Acinetobacter baumannii* genomes from Malaysia used to construct the phylogenetic tree

| **Strain ID** | **Location** | **Latitude** | **Longitude** | **Country** | **Year** | **ST (Oxford)** | **ST (Pasteur)** | **Global Clone** | **K locus** | **O locus** | **R3 Plasmid** | ***comM* intactness** |
| --- | --- | --- | --- | --- | --- | --- | --- | --- | --- | --- | --- | --- |
| 19053 | Kg. Sungai Pergam | 4.0471 | 103.2859 | Malaysia | 2017 | 3542 | 2832 | NA | KL121 | OCL2 | Present | Intact |
| 19055 | Kg. Sungai Pergam | 4.0471 | 103.2859 | Malaysia | 2017 | 3543 | 2114 | NA | KL96 | OCL13 |  | Intact |
| 19056 | Kg. Sungai Pergam | 4.0471 | 103.2859 | Malaysia | 2017 | 3415 | 2522 | NA | KL170 | OCL2 | Present | Intact |
| 19058 | Kg. Sungai Pergam | 4.0471 | 103.2859 | Malaysia | 2017 | 3541 | 2834 | NA | KL202 | OCL4 | Present | Intact |
| 19060 | Kg. Sungai Pergam | 4.0471 | 103.2859 | Malaysia | 2017 | 871 | 2700 | NA | KL112 | OCL6 |  | Truncated |
| 19061 | Kg. Sungai Pergam | 4.0471 | 103.2859 | Malaysia | 2017 | 3544 | 470 | NA | KL172 | OCL4 | Present | Intact |
| 19062 | Kg. Sungai Pergam | 4.0471 | 103.2859 | Malaysia | 2017 | 3545 | 2635 | NA | KL81 | OCL6 |  | Intact |
| 19063 | Kg. Sungai Pergam | 4.0471 | 103.2859 | Malaysia | 2017 | 3540 | 2833 | NA | KL183 | OCL2 | Present | Intact |
| 19064 | Kg. Sungai Pergam | 4.0471 | 103.2859 | Malaysia | 2017 | 585 | 10 | 8 | KL108 | OCL2 | Present | Intact |
| 11795 SAMN12769618 | Unknown | 4.2105 | 101.9758 | Malaysia | 2017 | 2098 | 46 | NA | KL28 | OCL1 |  | Intact |
| 13581 SAMN03174915 | Unknown | 4.2105 | 101.9758 | Malaysia | 2012 | - | 739 | NA | KL229 | OCL6 | Present | Intact |
| 13582 SAMN03174917 | Unknown | 4.2105 | 101.9758 | Malaysia | 2012 | 2596 | 374 | NA | KL50 | OCL6 |  | Intact |
| 13583 SAMN03174920 | Unknown | 4.2105 | 101.9758 | Malaysia | 2012 | - | 360 | NA | KL52 | OCL6 |  | Truncated |
| 18713 AC1532 | Unknown | 4.2105 | 101.9758 | Malaysia | 2015 | 229 | 25 | 7 | KL14 | OCL6 |  | Truncated |
| 18714 AB24 | Unknown | 4.2105 | 101.9758 | Malaysia | 2012 | 229 | 25 | 7 | KL14 | OCL6 |  | Truncated |
| 18837 CRE341 | Unknown | 4.2105 | 101.9758 | Malaysia | 2015 | 1947 | 25 | 7 | KL139 | OCL6 |  | Truncated |
| 2733 AC30 | Terengganu | 5.3283 | 103.1412 | Malaysia | 2011 | 195 | 2 | 2 | KL3 | OCL1 | Present | Truncated |
| 2754 AC12 | Terengganu | 5.3283 | 103.1412 | Malaysia | 2011 | 195 | 2 | 2 | KL3 | OCL1 | Present | Truncated |
| 2756 AC29 | Terengganu | 5.3283 | 103.1412 | Malaysia | 2011 | 195 | 2 | 2 | KL3 | OCL1 | Present | Truncated |
| 2942 341 | Unknown | 4.2105 | 101.9758 | Malaysia | 2013 | - | 2 | 2 | KL210 | OCL1 |  | Truncated |
| 4127 CRE341 2015 | Kuala Lumpur | 3.1319 | 101.6841 | Malaysia | 2015 | 1947 | 25 | 7 | KL139 | OCL6 | Present | Truncated |
| 4128 CRE449 2014 | Kuala Lumpur | 3.1319 | 101.6841 | Malaysia | 2014 | - | - | NA | KL3 | OCL1 | Present | Truncated |
| 6871 CRE449 | Pahang | 3.9743 | 102.4381 | Malaysia | 2014 | - | - | NA | KL3 | OCL1 | Present | Truncated |
| 7942 AC1839 | Terengganu | 5.3283 | 103.1412 | Malaysia | 2018 | - | - | NA | KL120 | OCL1 |  | Intact |
| 7943 AC1934 | Terengganu | 5.3283 | 103.1412 | Malaysia | 2019 | - | - | NA | KL176 | OCL5 |  | Intact |
| 9479 AB863 | Unknown | 4.2105 | 101.9758 | Malaysia | 2014 | - | 2 | 2 | KL210 | OCL1 | Present | Truncated |
| 9480 AB889 | Unknown | 4.2105 | 101.9758 | Malaysia | 2014 | - | 2 | 2 | KL3 | OCL1 | Present | Truncated |
| 9481 AB930 | Unknown | 4.2105 | 101.9758 | Malaysia | 2014 | - | 2 | 2 | KL3 | OCL1 | Present | Truncated |
| AB1 | Segamat | 2.5035 | 102.8208 | Malaysia | 2012 | - | 2 | 2 | KL3 | OCL1 | Present | Truncated |
| AB13 | Segamat | 2.5035 | 102.8208 | Malaysia | 2012 | - | 2 | 2 | KL3 | OCL1 | Present | Truncated |
| AB17 | Segamat | 2.5035 | 102.8208 | Malaysia | 2012 | 2006 | 1230 | NA | KL54 | OCL2 |  | Intact |
| AB18 | Segamat | 2.5035 | 102.8208 | Malaysia | 2012 | - | 2 | 2 | KL3 | OCL1 | Present | Truncated |
| AB2 | Segamat | 2.5035 | 102.8208 | Malaysia | 2012 | - | 2 | 2 | KL3 | OCL1 | Present | Truncated |
| AB20 | Segamat | 2.5035 | 102.8208 | Malaysia | 2012 | - | 2 | 2 | KL3 | OCL1 | Present | Truncated |
| AB21 | Segamat | 2.5035 | 102.8208 | Malaysia | 2012 | - | 2 | 2 | KL3 | OCL1 | Present | Truncated |
| AB22 | Segamat | 2.5035 | 102.8208 | Malaysia | 2012 | - | 2 | 2 | KL3 | OCL1 | Present | Truncated |
| AB23 | Segamat | 2.5035 | 102.8208 | Malaysia | 2012 | - | 2 | 2 | KL3 | OCL1 | Present | Truncated |
| AB4 | Segamat | 2.5035 | 102.8208 | Malaysia | 2012 | - | 2 | 2 | KL3 | OCL1 | Present | Truncated |
| AB5 | Segamat | 2.5035 | 102.8208 | Malaysia | 2012 | - | 2 | 2 | KL3 | OCL1 | Present | Truncated |
| AB6 | Segamat | 2.5035 | 102.8208 | Malaysia | 2012 | - | 2 | 2 | KL3 | OCL1 | Present | Truncated |
| AB8 | Segamat | 2.5035 | 102.8208 | Malaysia | 2012 | - | 2 | 2 | KL3 | OCL1 | Present | Truncated |
| AC1106 | Terengganu | 5.3283 | 103.1412 | Malaysia | 2011 | 1000 | 638 | NA | KL47 | OCL7 |  | Intact |
| AC1108 | Terengganu | 5.3283 | 103.1412 | Malaysia | 2011 | 195 | 2 | 2 | KL3 | OCL1 | Present | Truncated |
| AC1119 | Terengganu | 5.3283 | 103.1412 | Malaysia | 2011 | 207 | 1 | 1 | KL4 | OCL3 |  | Truncated |
| AC1123 | Terengganu | 5.3283 | 103.1412 | Malaysia | 2011 | 195 | 2 | 2 | KL3 | OCL1 | Present | Truncated |
| AC1138 | Terengganu | 5.3283 | 103.1412 | Malaysia | 2011 | 195 | 2 | 2 | KL3 | OCL1 | Present | Truncated |
| AC1140 | Terengganu | 5.3283 | 103.1412 | Malaysia | 2011 | 938 | 2 | 2 | KL210 | OCL1 | Present | Truncated |
| AC1201 | Terengganu | 5.3283 | 103.1412 | Malaysia | 2012 | 195 | 2 | 2 | KL3 | OCL1 | Present | Truncated |
| AC1202 | Terengganu | 5.3283 | 103.1412 | Malaysia | 2012 | 195 | 2 | 2 | KL3 | OCL1 | Present | Truncated |
| AC1204 | Terengganu | 5.3283 | 103.1412 | Malaysia | 2012 | 195 | 2 | 2 | KL3 | OCL1 | Present | Truncated |
| AC1205 | Terengganu | 5.3283 | 103.1412 | Malaysia | 2012 | 195 | 2 | 2 | KL3 | OCL1 | Present | Truncated |
| AC1206 | Terengganu | 5.3283 | 103.1412 | Malaysia | 2012 | 195 | 2 | 2 | KL3 | OCL1 | Present | Truncated |
| AC1208 | Terengganu | 5.3283 | 103.1412 | Malaysia | 2012 | 195 | 2 | 2 | KL3 | OCL1 | Present | Truncated |
| AC1213 | Terengganu | 5.3283 | 103.1412 | Malaysia | 2012 | 195 | 2 | 2 | KL3 | OCL1 | Present | Truncated |
| AC1217 | Terengganu | 5.3283 | 103.1412 | Malaysia | 2012 | 2006 | 1230 | NA | KL54 | OCL2 |  | Intact |
| AC1218 | Terengganu | 5.3283 | 103.1412 | Malaysia | 2012 | 195 | 2 | 2 | KL3 | OCL1 | Present | Truncated |
| AC1220 | Terengganu | 5.3283 | 103.1412 | Malaysia | 2012 | 195 | 2 | 2 | KL3 | OCL1 | Present | Truncated |
| AC1221 | Terengganu | 5.3283 | 103.1412 | Malaysia | 2012 | 195 | 2 | 2 | KL3 | OCL1 | Present | Truncated |
| AC1222 | Terengganu | 5.3283 | 103.1412 | Malaysia | 2012 | 195 | 2 | 2 | KL3 | OCL1 | Present | Truncated |
| AC1223 | Terengganu | 5.3283 | 103.1412 | Malaysia | 2012 | 195 | 2 | 2 | KL3 | OCL1 | Present | Truncated |
| AC1224 | Terengganu | 5.3283 | 103.1412 | Malaysia | 2012 | 229 | 25 | 7 | KL14 | OCL6 |  | Truncated |
| AC15101 | Segamat | 2.5035 | 102.8208 | Malaysia | 2015 | - | 2 | 2 | KL2 | OCL1 | Present | Truncated |
| AC15139 | Segamat | 2.5035 | 102.8208 | Malaysia | 2015 | 1201 | 721 | NA | KL81 | OCL6 |  | Intact |
| AC15163 | Segamat | 2.5035 | 102.8208 | Malaysia | 2015 | - | 2 | 2 | KL210 | OCL1 | Present | Truncated |
| AC15190 | Segamat | 2.5035 | 102.8208 | Malaysia | 2015 | 1418 | 164 | NA | KL47 | OCL5 |  | Truncated |
| AC1520 | Segamat | 2.5035 | 102.8208 | Malaysia | 2015 | - | 2 | 2 | KL2 | OCL1 | Present | Truncated |
| AC1521 | Segamat | 2.5035 | 102.8208 | Malaysia | 2015 | - | 2 | 2 | KL2 | OCL1 | Present | Truncated |
| AC1522 | Terengganu | 5.3283 | 103.1412 | Malaysia | 2015 | 195 | 2 | 2 | KL3 | OCL1 | Present | Truncated |
| AC1533 | Segamat | 2.5035 | 102.8208 | Malaysia | 2015 | - | 2 | 2 | KL3 | OCL1 | Present | Truncated |
| AC1538 | Segamat | 2.5035 | 102.8208 | Malaysia | 2015 | - | 2 | 2 | KL210 | OCL1 | Present | Truncated |
| AC1556 | Terengganu | 5.3283 | 103.1412 | Malaysia | 2015 | 2021 | 504 | NA | KL118 | UK |  | Intact |
| AC1557 | Terengganu | 5.3283 | 103.1412 | Malaysia | 2015 | 208 | 2 | 2 | KL2 | OCL1 | Present | Truncated |
| AC156 | Terengganu | 5.3283 | 103.1412 | Malaysia | 2015 | 192 | 2 | 2 | KL2 | OCL1 | Present | Truncated |
| AC1560 | Segamat | 2.5035 | 102.8208 | Malaysia | 2015 | 2022 | 77 | NA | KL33 | OCL2 |  | Intact |
| AC1561 | Segamat | 2.5035 | 102.8208 | Malaysia | 2015 | 1320 | 265 | NA | KL109 | OCL4 |  | Intact |
| AC1601 | Terengganu | 5.3283 | 103.1412 | Malaysia | 2016 | NA | 2 | 2 | UK | OCL1 | Present | Truncated |
| AC1602 | Terengganu | 5.3283 | 103.1412 | Malaysia | 2016 | 938 | 2 | 2 | KL210 | OCL1 | Present | Truncated |
| AC1607 | Terengganu | 5.3283 | 103.1412 | Malaysia | 2016 | 1980 | 1093 | NA | UK | OCL2 |  | Intact |
| AC1612 | Terengganu | 5.3283 | 103.1412 | Malaysia | 2016 | 207 | 1 | 1 | KL4 | OCL3 |  | Truncated |
| AC1613 | Terengganu | 5.3283 | 103.1412 | Malaysia | 2016 | 547 | 2 | 2 | KL14 | OCL1 | Present | Truncated |
| AC1617 | Terengganu | 5.3283 | 103.1412 | Malaysia | 2016 | 942 | 267 | NA | KL9 | OCL2 |  | Intact |
| AC1619 | Terengganu | 5.3283 | 103.1412 | Malaysia | 2016 | 1209 | 727 | NA | KL47 | OCL6 |  | Intact |
| AC1623 | Terengganu | 5.3283 | 103.1412 | Malaysia | 2016 | 2090 | 1147 | NA | KL9 | OCL2 |  | Intact |
| AC1633 | Terengganu | 5.3283 | 103.1412 | Malaysia | 2016 | 2089 | 126 | NA | KL14 | OCL6 |  | Intact |
| AC1638 | Terengganu | 5.3283 | 103.1412 | Malaysia | 2016 | 2024 | 1102 | NA | UK | OCL6 |  | Truncated |
| AC1639 | Terengganu | 5.3283 | 103.1412 | Malaysia | 2016 | 234 | 164 | 11 | KL106 | OCL8 |  | Intact |
| AC1701 | Terengganu | 5.3283 | 103.1412 | Malaysia | 2017 | 514 | 103 | NA | KL24 | OCL7 |  | Intact |
| AC1702 | Terengganu | 5.3283 | 103.1412 | Malaysia | 2017 | 451 | 2 | 2 | KL120 | OCL1 | Present | Truncated |
| AC1704 | Terengganu | 5.3283 | 103.1412 | Malaysia | 2017 | 208 | 2 | 2 | KL2 | OCL1 | Present | Truncated |
| AC1705 | Terengganu | 5.3283 | 103.1412 | Malaysia | 2017 | 208 | 2 | 2 | KL2 | OCL1 | Present | Truncated |
| AC1709 | Terengganu | 5.3283 | 103.1412 | Malaysia | 2017 | 547 | 2 | 2 | KL14 | OCL1 | Present | Truncated |
| AC1712 | Terengganu | 5.3283 | 103.1412 | Malaysia | 2017 | 547 | 2 | 2 | KL14 | OCL1 | Present | Truncated |
| AC1713 | Terengganu | 5.3283 | 103.1412 | Malaysia | 2017 | 3387 | 1131 | NA | KL26 | OCL9 |  | Intact |
| AC1716 | Terengganu | 5.3283 | 103.1412 | Malaysia | 2017 | 451 | 2 | 2 | KL120 | OCL1 | Present | Truncated |
| AC1718 | Terengganu | 5.3283 | 103.1412 | Malaysia | 2017 | 208 | 2 | 2 | KL2 | OCL1 | Present | Truncated |
| AC1729 | Terengganu | 5.3283 | 103.1412 | Malaysia | 2017 | 195 | 2 | 2 | KL3 | OCL1 | Present | Truncated |
| AC1732 | Terengganu | 5.3283 | 103.1412 | Malaysia | 2017 | 231 | 1 | 1 | KL1 | OCL1 |  | Truncated |
| AC1738 | Terengganu | 5.3283 | 103.1412 | Malaysia | 2017 | 2199 | 1 | 1 | KL4 | OCL3 | Present | Truncated |
| AC1739 | Terengganu | 5.3283 | 103.1412 | Malaysia | 2017 | 547 | 2 | 2 | KL14 | OCL1 | Present | Truncated |
| AC1750 | Terengganu | 5.3283 | 103.1412 | Malaysia | 2017 | 547 | 2 | 2 | KL14 | OCL1 | Present | Truncated |
| AC1752 | Terengganu | 5.3283 | 103.1412 | Malaysia | 2017 | 547 | 2 | 2 | KL14 | OCL1 | Present | Truncated |
| AC1759 | Terengganu | 5.3283 | 103.1412 | Malaysia | 2017 | 3388 | 729 | NA | KL6 | OCL2 |  | Intact |
| AC1763 | Terengganu | 5.3283 | 103.1412 | Malaysia | 2017 | 684 | 2 | 2 | KL155 | OCL1 | Present | Truncated |
| AC1779 | Terengganu | 5.3283 | 103.1412 | Malaysia | 2017 | 1418 | 164 | 11 | KL47 | OCL5 |  | Truncated |
| AC1780 | Terengganu | 5.3283 | 103.1412 | Malaysia | 2017 | 195 | 2 | 2 | KL32 | OCL1 | Present | Truncated |
| AC1794 | Terengganu | 5.3283 | 103.1412 | Malaysia | 2017 | 3389 | 960 | NA | KL26 | OCL11 |  | Intact |
| AC1795 | Terengganu | 5.3283 | 103.1412 | Malaysia | 2017 | 684 | 2 | 2 | KL155 | OCL1 | Present | Truncated |
| AC18102 | Terengganu | 5.3283 | 103.1412 | Malaysia | 2018 | 1980 | 1093 | NA | KL49 | OCL2 |  | Intact |
| AC18103 | Terengganu | 5.3283 | 103.1412 | Malaysia | 2018 | 684 | 2 | 2 | KL155 | OCL1 | Present | Truncated |
| AC18111 | Terengganu | 5.3283 | 103.1412 | Malaysia | 2018 | 684 | 2 | 2 | KL155 | OCL1 | Present | Truncated |
| AC1813 | Segamat | 2.5035 | 102.8208 | Malaysia | 2013 | - | 2 | 2 | KL32 | OCL1 | Present | Truncated |
| AC1828 | Segamat | 2.5035 | 102.8208 | Malaysia | 2018 | - | 2 | 2 | KL32 | OCL1 |  | Truncated |
| AC1829 | Segamat | 2.5035 | 102.8208 | Malaysia | 2018 | - | 2 | 2 | KL32 | OCL1 | Present | Truncated |
| AC1830 | Terengganu | 5.3283 | 103.1412 | Malaysia | 2018 | 195 | 2 | 2 | KL32 | OCL1 | Present | Truncated |
| AC1831 | Terengganu | 5.3283 | 103.1412 | Malaysia | 2018 | 195 | 2 | 2 | KL32 | OCL1 | Present | Truncated |
| AC1836 | Terengganu | 5.3283 | 103.1412 | Malaysia | 2018 | 195 | 2 | 2 | KL32 | OCL1 | Present | Truncated |
| AC1845 | Terengganu | 5.3283 | 103.1412 | Malaysia | 2018 | 684 | 2 | 2 | KL155 | OCL1 | Present | Truncated |
| AC1847 | Terengganu | 5.3283 | 103.1412 | Malaysia | 2018 | 1418 | 164 | NA | KL47 | OCL5 |  | Truncated |
| AC1859 | Terengganu | 5.3283 | 103.1412 | Malaysia | 2018 | 3390 | 79 | 5 | KL32 | OCL10 | Present | Truncated |
| AC1866 | Terengganu | 5.3283 | 103.1412 | Malaysia | 2018 | 1418 | 164 | 11 | KL47 | OCL5 |  | Truncated |
| AC1873 | Terengganu | 5.3283 | 103.1412 | Malaysia | 2018 | 195 | 2 | 2 | KL32 | OCL1 | Present | Truncated |
| AC1882 | Terengganu | 5.3283 | 103.1412 | Malaysia | 2018 | 195 | 2 | 2 | KL32 | OCL1 | Present | Truncated |
| AC1883 | Terengganu | 5.3283 | 103.1412 | Malaysia | 2018 | 753 | 1620 | NA | KL112 | OCL9 |  | Intact |
| AC1884 | Terengganu | 5.3283 | 103.1412 | Malaysia | 2018 | 684 | 2 | 2 | KL155 | OCL1 | Present | Truncated |
| AC1890 | Terengganu | 5.3283 | 103.1412 | Malaysia | 2018 | 547 | 2 | 2 | KL14 | OCL1 | Present | Truncated |
| AC1891 | Terengganu | 5.3283 | 103.1412 | Malaysia | 2018 | 1088 | 338 | NA | UK | UK |  | Intact |
| AC1898 | Terengganu | 5.3283 | 103.1412 | Malaysia | 2018 | 195 | 2 | 2 | KL32 | OCL1 | Present | Truncated |
| AC19 | Segamat | 2.5035 | 102.8208 | Malaysia | 2011 | - | 1 | 1 | KL4 | OCL3 |  | Truncated |
| AC1902 | Terengganu | 5.3283 | 103.1412 | Malaysia | 2019 | 207 | 1 | 1 | KL4 | OCL3 |  | Truncated |
| AC19106 | Terengganu | 5.3283 | 103.1412 | Malaysia | 2019 | 514 | 103 | NA | KL24 | OCL7 |  | Truncated |
| AC19110 | Terengganu | 5.3283 | 103.1412 | Malaysia | 2019 | 195 | 2 | 2 | KL32 | OCL1 | Present | Truncated |
| AC19111 | Terengganu | 5.3283 | 103.1412 | Malaysia | 2019 | 1418 | 164 | 11 | KL47 | OCL5 |  | Truncated |
| AC19112 | Terengganu | 5.3283 | 103.1412 | Malaysia | 2019 | 2294 | 164 | 11 | KL111 | OCL8 |  | Intact |
| AC19113 | Terengganu | 5.3283 | 103.1412 | Malaysia | 2019 | 195 | 2 | 2 | KL32 | OCL1 | Present | Truncated |
| AC19117 | Terengganu | 5.3283 | 103.1412 | Malaysia | 2019 | 195 | 2 | 2 | KL32 | OCL1 | Present | Truncated |
| AC19128 | Terengganu | 5.3283 | 103.1412 | Malaysia | 2019 | 585 | 10 | 8 | KL108 | OCL2 |  | Intact |
| AC19135 | Terengganu | 5.3283 | 103.1412 | Malaysia | 2019 | 3391 | 132 | 10 | UK | OCL6 |  | Intact |
| AC19142 | Terengganu | 5.3283 | 103.1412 | Malaysia | 2019 | 208 | 2 | 2 | KL2 | OCL1 | Present | Truncated |
| AC19145 | Terengganu | 5.3283 | 103.1412 | Malaysia | 2019 | 208 | 2 | 2 | KL2 | OCL1 | Present | Truncated |
| AC1919 | Terengganu | 5.3283 | 103.1412 | Malaysia | 2019 | 684 | 2 | 2 | KL155 | OCL1 | Present | Truncated |
| AC1930 | Terengganu | 5.3283 | 103.1412 | Malaysia | 2019 | 451 | 2 | 2 | KL120 | OCL1 | Present | Truncated |
| AC1932 | Terengganu | 5.3283 | 103.1412 | Malaysia | 2019 | 1496 | 1405 | 10 | KL30 | OCL5 |  | Intact |
| AC1940 | Terengganu | 5.3283 | 103.1412 | Malaysia | 2019 | 684 | 2 | 2 | KL155 | OCL1 | Present | Truncated |
| AC1950 | Terengganu | 5.3283 | 103.1412 | Malaysia | 2019 | 684 | 2 | 2 | UK | OCL1 | Present | Truncated |
| AC1956 | Terengganu | 5.3283 | 103.1412 | Malaysia | 2019 | 447 | 10 | 8 | KL49 | OCL2 |  | Intact |
| AC1957 | Terengganu | 5.3283 | 103.1412 | Malaysia | 2019 | 195 | 2 | 2 | KL32 | OCL1 | Present | Truncated |
| AC1989 | Terengganu | 5.3283 | 103.1412 | Malaysia | 2019 | 208 | 2 | 2 | KL2 | OCL1 | Present | Truncated |
| AC2009 | Terengganu | 5.3283 | 103.1412 | Malaysia | 2020 | 3392 | 139 | NA | KL116 | OCL5 |  | Intact |
| AC2010 | Terengganu | 5.3283 | 103.1412 | Malaysia | 2020 | 2271 | 1112 | NA | KL51 | OCL6 |  | Truncated |
| AC2013 | Terengganu | 5.3283 | 103.1412 | Malaysia | 2020 | 942 | 267 | NA | KL9 | OCL2 |  | Truncated |
| AC2014 | Terengganu | 5.3283 | 103.1412 | Malaysia | 2020 | 3393 | 142 | NA | KL59 | OCL5 |  | Intact |
| AC2015 | Terengganu | 5.3283 | 103.1412 | Malaysia | 2020 | 195 | 2 | 2 | KL32 | OCL1 | Present | Truncated |
| AC2018 | Terengganu | 5.3283 | 103.1412 | Malaysia | 2020 | 195 | 2 | 2 | KL32 | OCL1 | Present | Truncated |
| AC2024 | Terengganu | 5.3283 | 103.1412 | Malaysia | 2020 | 208 | 2 | 2 | KL2 | OCL1 | Present | Truncated |
| AC2025 | Terengganu | 5.3283 | 103.1412 | Malaysia | 2020 | 585 | 10 | 8 | KL108 | OCL2 |  | Intact |
| AC2028 | Terengganu | 5.3283 | 103.1412 | Malaysia | 2020 | 2827 | 2177 | NA | KL106 | OCL8 |  | Intact |
| AC2029 | Terengganu | 5.3283 | 103.1412 | Malaysia | 2020 | 208 | 2 | 2 | KL2 | OCL1 | Present | Truncated |
| AC2034 | Terengganu | 5.3283 | 103.1412 | Malaysia | 2020 | 208 | 2 | 2 | KL2 | OCL1 | Present | Truncated |
| AC2035 | Terengganu | 5.3283 | 103.1412 | Malaysia | 2020 | 208 | 2 | 2 | KL2 | OCL1 | Present | Truncated |
| AC2036 | Terengganu | 5.3283 | 103.1412 | Malaysia | 2020 | 208 | 2 | 2 | KL2 | OCL1 | Present | Truncated |
| AC2037 | Terengganu | 5.3283 | 103.1412 | Malaysia | 2020 | 208 | 2 | 2 | KL2 | OCL1 | Present | Truncated |
| AC2041 | Terengganu | 5.3283 | 103.1412 | Malaysia | 2020 | 884 | 113 | 7 | KL10 | OCL5 |  | Intact |
| AC2043 | Terengganu | 5.3283 | 103.1412 | Malaysia | 2020 | 1000 | 638 | NA | KL47 | OCL7 |  | Truncated |
| AC2044 | Terengganu | 5.3283 | 103.1412 | Malaysia | 2020 | 195 | 2 | 2 | KL32 | OCL1 |  | Truncated |
| AC2045 | Terengganu | 5.3283 | 103.1412 | Malaysia | 2020 | 1324 | 374 | NA | KL50 | OCL2 |  | Truncated |
| AC2046 | Terengganu | 5.3283 | 103.1412 | Malaysia | 2020 | 208 | 2 | 2 | KL2 | OCL1 | Present | Truncated |
| AC23 | Segamat | 2.5035 | 102.8208 | Malaysia | 2011 | - | 2 | 2 | KL3 | OCL1 | Present | Truncated |
| AC38 | Segamat | 2.5035 | 102.8208 | Malaysia | 2011 | - | 2 | 2 | KL3 | OCL1 | Present | Truncated |
| AC40 | Segamat | 2.5035 | 102.8208 | Malaysia | 2011 | - | 2 | 2 | KL210 | OCL1 | Present | Truncated |
| AC6 | Segamat | 2.5035 | 102.8208 | Malaysia | 2011 | 1000 | 638 | NA | KL47 | OCL7 |  | Intact |
| AC8 | Segamat | 2.5035 | 102.8208 | Malaysia | 2011 | - | 2 | 2 | KL3 | OCL1 |  | Truncated |
| ATCC BAA1605 | Segamat | 2.5035 | 102.8208 | Malaysia | 2020 | - | 1 | 1 | KL15 | OCL3 | Present | Truncated |
| C-102 | Segamat | 2.5035 | 102.8208 | Malaysia | 2018 | 2235 | 2108 |  | KL223 | OCL5 |  | Intact |
| C-15 | Segamat | 2.5035 | 102.8208 | Malaysia | 2018 | 2230 | 216 | NA | KL75 | OCL6 |  | Intact |
| C-28 | Segamat | 2.5035 | 102.8208 | Malaysia | 2018 | 1463 | 284 | 10 | KL223 | OCL13 |  | Intact |
| C-39 | Segamat | 2.5035 | 102.8208 | Malaysia | 2018 | 485 | 338 | NA | KL197 | OCL14 |  | Intact |
| C-55 | Segamat | 2.5035 | 102.8208 | Malaysia | 2018 | 503 | 336 | NA | KL125 | OCL5 |  | Intact |
| C-59 | Segamat | 2.5035 | 102.8208 | Malaysia | 2018 | 2236 | - | NA | KL32 | OCL3 |  | Intact |
| C-61 | Segamat | 2.5035 | 102.8208 | Malaysia | 2018 | 2232 | 1411 | 10 | KL52 | OCL6 |  | Intact |
| C-64 | Segamat | 2.5035 | 102.8208 | Malaysia | 2018 | 1912 | 331 | NA | KL61 | OCL6 |  | Intact |
| C-72 | Segamat | 2.5035 | 102.8208 | Malaysia | 2018 | 128 | 49 | NA | KL11 | OCL8 |  | Truncated |
| C-95 | Segamat | 2.5035 | 102.8208 | Malaysia | 2018 | 2234 | - | NA | KL47 | OCL6 |  | Intact |
| C-98 | Segamat | 2.5035 | 102.8208 | Malaysia | 2018 | 231 | 1 | 1 | KL1 | OCL1 |  | Intact |
| H-10112 | Segamat | 2.5035 | 102.8208 | Malaysia | 2018 | 684 | 2 | 2 | KL155 | OCL1 | Present | Truncated |
| H-10156 | Segamat | 2.5035 | 102.8208 | Malaysia | 2018 | 208 | 2 | 2 | KL2 | OCL1 | Present | Truncated |
| H-10299 | Segamat | 2.5035 | 102.8208 | Malaysia | 2018 | 2238 | - |  | KL97 | OCL4 |  | Intact |
| H-10858 | Segamat | 2.5035 | 102.8208 | Malaysia | 2018 | 208 | 2 | 2 | KL2 | OCL1 | Present | Truncated |
| H-11553 | Segamat | 2.5035 | 102.8208 | Malaysia | 2018 | 447 | 10 | 8 | KL49 | OCL2 |  | Intact |
| H-11699 | Segamat | 2.5035 | 102.8208 | Malaysia | 2018 | 208 | 2 | 2 | KL2 | OCL1 |  | Truncated |
| H-52446 | Segamat | 2.5035 | 102.8208 | Malaysia | 2018 | 208 | 2 | 2 | KL2 | OCL1 | Present | Truncated |
| H-6657 | Segamat | 2.5035 | 102.8208 | Malaysia | 2018 | 684 | 2 | 2 | KL155 | OCL1 | Present | Truncated |
| H-6668 | Segamat | 2.5035 | 102.8208 | Malaysia | 2018 | 2237 | 459 | 10 | KL128 | OCL2 |  | Intact |
| H-7940 | Segamat | 2.5035 | 102.8208 | Malaysia | 2018 | 208 | 2 | 2 | KL2 | OCL1 | Present | Truncated |
| H-79532 | Segamat | 2.5035 | 102.8208 | Malaysia | 2018 | 2241 | 23 | 8 | KL8 | OCL2 |  | Intact |
| H-80330 | Segamat | 2.5035 | 102.8208 | Malaysia | 2018 | 208 | 2 | 2 | KL2 | OCL1 | Present | Truncated |
| H-80359 | Segamat | 2.5035 | 102.8208 | Malaysia | 2018 | 547 | 2 | 2 | KL14 | OCL1 | Present | Truncated |
| H-80361 | Segamat | 2.5035 | 102.8208 | Malaysia | 2018 | 547 | 2 | 2 | KL14 | OCL1 | Present | Truncated |
| H-80400 | Segamat | 2.5035 | 102.8208 | Malaysia | 2018 | 207 | 2 | 2 | KL2 | OCL1 | Present | Truncated |
| K09-14 | Segamat | 2.5035 | 102.8208 | Malaysia | 2017 | 2098 | 46 |  | KL28 | OCL1 |  | Intact |

**APPENDIX 2:** List of insertion sequence (IS) elements found in the Orang Asli *A. baumannii* genomes

| ***A. baumannii* isolate ID** | **IS elements (family)** |
| --- | --- |
| 19053 | ISAba43 (ISL3), ISAba57 (IS3), ISAba40 (IS3) |
| 19055 | ISAba43 (ISL3) |
| 19056 | ISAba43 (ISL3) |
| 19058 | ISAba43 (ISL3) |
| 19060 | ISAba43 (ISL3) |
| 19061 | ISAba43 (ISL3) |
| 19062 | ISAba43 (ISL3), ISAba68 (IS5), IS1006 (IS91), ISAba44 (IS630) |
| 19063 | ISAba43 (ISL3) |
| 19064 | ISAba43 (ISL3), ISAba63 (IS3) |

**APPENDIX 3:** List of plasmids identified from the genomes of the Orang Asli *A. baumannii* isolates.

| ***A. baumannii* strain ID** | **Plasmids (*n*)** | **Plasmid name** | **Location** | **Size (bp)** | **Rep family** | **Coverage (x)** |
| --- | --- | --- | --- | --- | --- | --- |
| 19053 | 2 | p19053a | contig29 | 2,178 | R1-T6 | 198 |
|  |  | p19053b | contig20 | 8,837 | R3-T5 | 20.1 |
|  |  |  |  |  |  |  |
| 19055 | 0 | - | - | - | - | - |
|  |  |  |  |  |  |  |
| 19056 | 1 | p19056 | contig15 | 7,146 | R3-T5 | 81 |
|  |  |  |  |  |  |  |
| 19058 | 1 | p19058 | contig24 | 7,701 | R3-T13 | 29.7 |
|  |  |  |  |  |  |  |
| 19060 | 1 | p19060 | contig56 | 14,883 | R3-T26 | 26.7 |
|  |  |  |  |  |  |  |
| 19061 | 1 | p19061 | contig14 | 7,146 | R3-T5 | 39.2 |
|  |  |  |  |  |  |  |
| 19062 | 2 | p19062a | contig51 | 8,039 | R3-T64 | 562.6 |
|  |  | p19062b | contig51 | 10,209 | R3-T13 | 562.6 |
|  |  |  |  |  |  |  |
| 19063 | 1 | p19063 | contig16 | 7,752 | R3-T13 | 330.2 |
|  |  |  |  |  |  |  |
| 19064 | 2 | p19064a | contig34, 35 | 7,416 | R3-T5 | 201.7 |
|  |  | p19064b | contig27 | 19,328 | R3-T27 | 20.9 |
|  |  |  |  |  |  |  |

**APPENDIX 4:** Linear genetic maps of all plasmids identified in the Orang Asli community *A. baumannii* isolates. Majority of the plasmids were of the Rep_3 family with only plasmid, p19053a, belonging to the Rep_1 family. The *rep*-encoded replication initiation protein gene is depicted as blue-colored arrows with its Rep type labelled while its downstream gene, recently designated *orfX* (Lam et al., 2023), is shown as light blue arrows. White colored arrows represent genes encoding hypothetical proteins. Other colored arrows are as in the legends to **Figures 4** and **5** in the main text. p*dif* sites are depicted as green (XerC/D) and red (XerD/C) crosses. Note that plasmid p19064a is a composite of two contigs that were stitched together as signified by the needle and thread icon above its linear map.


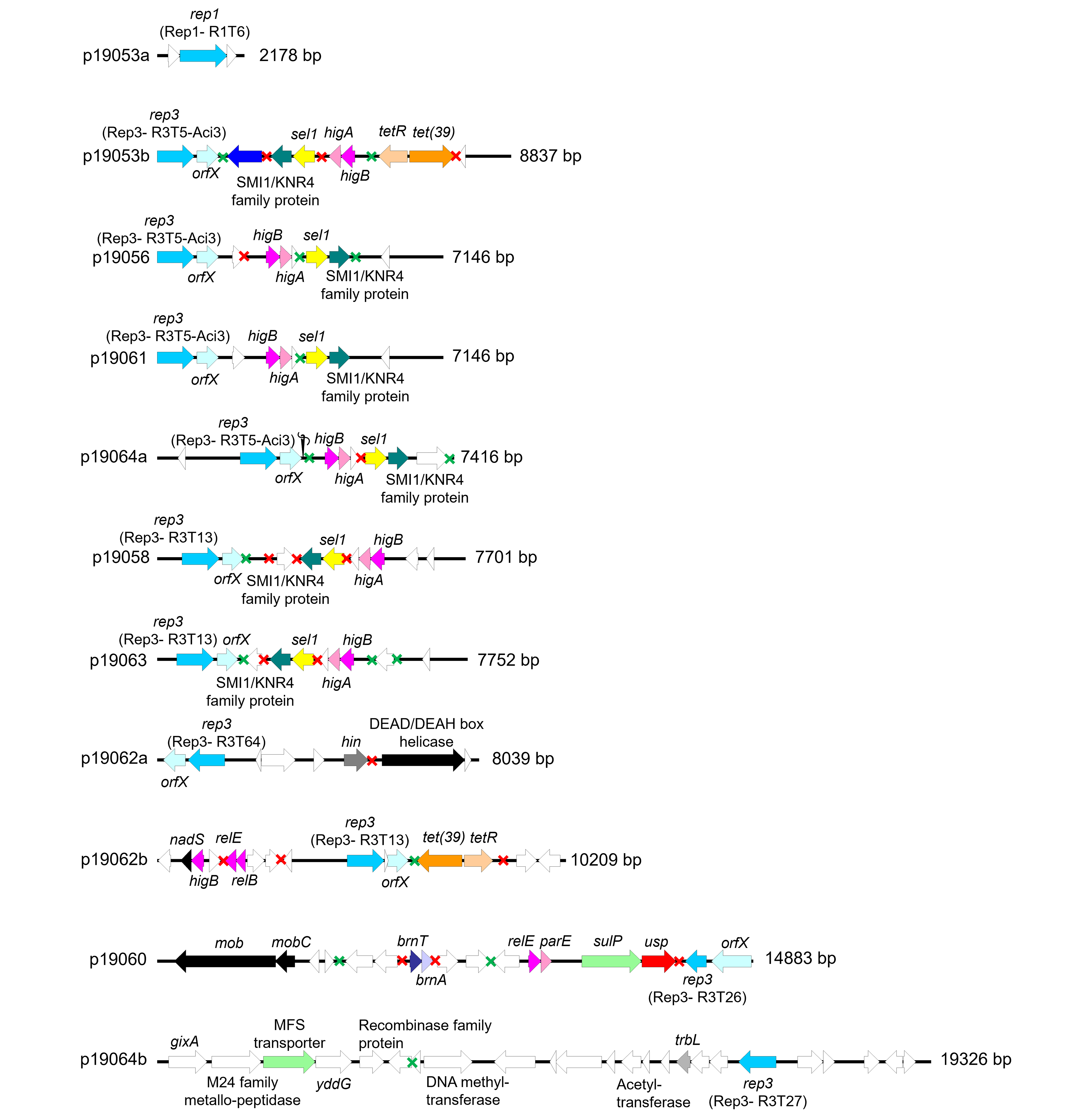


**APPENDIX 5:** List of p*dif* sequences identified in the Orang Asli *A. baumannii* plasmids

| **Plasmid** | **Start** | **End** | **Sequence (5'🡪3')*** | **Type^§^** |
| --- | --- | --- | --- | --- |
| p19053a | ND^#^ | ND^#^ | - | - |
| p19053b | 1696 | 1723 | ATTTCGTATAA GGCGTA TTATGTTAATT | C\|D |
|  | 2727 | 2754 | ATTTAACATAA TGGGCG TTATATGAAAT | D\|C |
|  | 4048 | 4075 | ATTTAACATAG AAACTC TTATGCGAATC | D\|C |
|  | 5445 | 5472 | ATTTCGGATAA CGCCCA TTATGTTAAAT | C\|D |
|  | 7474 | 7501 | AATTAACATAA TACGCC TTATGCGAAGC | D\|C |
| p19056 | 2187 | 2214 | ATTTAACATAA TGGGCG TTATCCGAAAT | D\|C |
|  | 3582 | 3609 | GATTCGCATAA GAGTTT CTATGTTAAAT | C\|D |
|  | 4902 | 4929 | ATTTCATATAA CGCCCA TTATGTTAAAT | C\|D |
| p19058 | 2293 | 2320 | ATTTCGTATAA GGTGTA TTATGTTAATT | C\|D |
|  | 2945 | 2972 | ATTTAACATAA AATCTC TTATTCGAAAT | D\|C |
|  | 3466 | 3493 | ATTTAACATAA TGGGCG TTATATGAAAT | D\|C |
|  | 4786 | 4813 | ATTTAACATAG AAACTC TTATGCGAATC | D\|C |
| p19060 | 4641 | 4668 | ATTTCGTATAA GGCGTA TTATGTTAATT | C\|D |
|  | 6088 | 6115 | ATTTAACATAA TGGGCG TTATACGAAAC | D\|C |
|  | 6933 | 6960 | GTATAAGGTGT ATTATG TTAATTTTAGA | D\|C |
|  | 8372 | 8399 | GCTTCACATAA GAGATT TTATGTTAAAT | C\|D |
|  | 13010 | 13037 | AATTAACATAA TACACC TTATACGAAAT | D\|C |
| p19061 | 3582 | 3609 | GATTCGCATAA GAGTTT CTATGTTAAAT | C\|D |
| p19062a | 2685 | 2712 | ATTTAACATAA AATCTC TTATGTGAAGC | D\|C |
| p19062b | 1623 | 1650 | AATTAACATAA TACACC TTATACGAAGG | D\|C |
|  | 3109 | 3136 | TCTAAAATTAA CATAAT ACACCTTATAC | D\|C |
|  | 6421 | 6448 | GTATAAGGTGT ATTATG TTAATTTTAGA | C\|D |
|  | 8445 | 8472 | ATTTAACATAA TGGGCG TTATCCGAAAT | D\|C |
| p19063 | 2160 | 2187 | ATTTCGTATAA GGTGTA TTATGTTAATT | C\|D |
|  | 2701 | 2728 | ATTTAACATAA TGGGCG TTATATGAAAT | D\|C |
|  | 4021 | 4048 | ATTTAACATAG AAACTC TTATGCGAATC | D\|C |
|  | 5413 | 5440 | ATTTCGGATAA CGCCCA TTATGTTAAAT | C\|D |
|  | 5934 | 5961 | ATTTCGAATAA GAGATT TTATGTTAAAT | C\|D |
| p19064a | 3750 | 3777 | ATTTCGTATAA GGCGTA TTATGTTAATT | C\|D |
|  | 5992 | 6019 | ATTTAACATAG AAACTC TTATGCGAATC | D\|C |
|  | 7387 | 7414 | ATTTCGGATAA CGCCCA TTATGTTAAAT | C\|D |
| p19064b | 5705 | 5732 | GCTTCACATAA GAGATT TTATGTTAAAT | C\|D |

*p*dif* sites are presented with underlined fonts representing the 6 bp spacer that is flanked by the XerC/XerD binding sites.

^§^C|D refers to the XerC-spacer-XerD orientation while D|C refers to the XerD-spacer-XerC orientation of the p*dif* site

^#^ND = not detected
